# Discrete Brush Polymers Enhance ^19^F MRI Performance through Architectural Precision

**DOI:** 10.1021/jacs.5c00938

**Published:** 2025-05-01

**Authors:** Nduka D. Ogbonna, Parikshit Guragain, Venkatesh Mayandi, Cyrus Sadrinia, Raman Danrad, Seetharama Jois, Jimmy Lawrence

**Affiliations:** † Department of Chemical Engineering, Louisiana State University, Baton Rouge, Louisiana 70803, United States; ‡ Department of Pathological Sciences, School of Veterinary Medicine, Louisiana State University, Baton Rouge, Louisiana 70803, United States; § Department of Radiology, School of Medicine, Louisiana State University Health, New Orleans, Louisiana 70112, United States

## Abstract

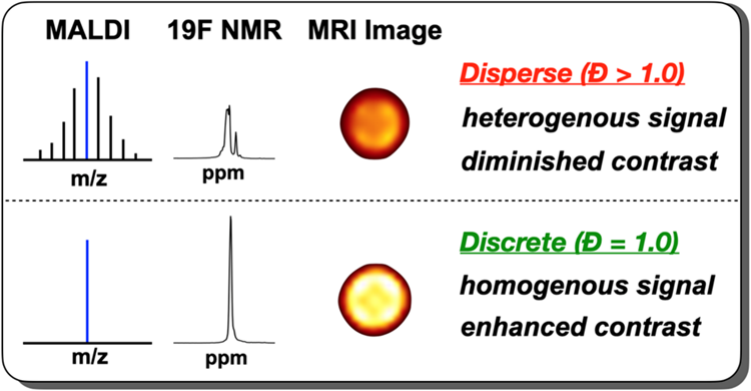

The development of metal-free magnetic resonance imaging (MRI) agents demands precise control over molecular architecture to achieve optimal performance. Current fluorine-based contrast agents rely on maximizing fluorine content (>20 wt %) for sensitivity, requiring extensive solubilizing groups that lead to signal-diminishing aggregation. Here we show that discrete brush polymers (*Đ* = 1.0) with precise backbone lengths and a single terminal fluorine group achieve superior imaging performance through architectural control rather than high fluorine content. This design prevents both intra- and intermolecular fluorine aggregation while maintaining high aqueous solubility, enabling sharper signals and higher sensitivity than conventional systems despite containing less than 7 wt % fluorine. Systematic investigation reveals how backbone length controls fluorine mobility and signal generation, establishing clear structure–property relationships previously obscured by molecular heterogeneity. This work demonstrates how precise architectural control can enhance functional performance beyond traditional approaches, providing new strategies for designing imaging materials.

## Introduction

The precise control of macromolecular architecture represents a fundamental challenge in polymer science, particularly for complex nonlinear topologies where molecular heterogeneity obscures structure–property relationships.^1−8^ While nature demonstrates the power of architectural precision in materials like aggrecan,^9,10^ achieving similar control in synthetic systems has remained elusive. Recent advances combining controlled polymerization with library isolation of discrete polymers using chromatographic separation (termed CLIP) have enabled access to discrete structures (*Đ* = 1.0), providing unprecedented insights into how molecular precision governs material performance.^11−15^ The combination of architectural control and scalability offered by the CLIP strategy enables the development of new functional materials for applications requiring a high degree of precision, exemplified by metal-free magnetic resonance imaging (MRI) contrast agents where signal quality depends critically on molecular uniformity.^16^

**Scheme 1:**
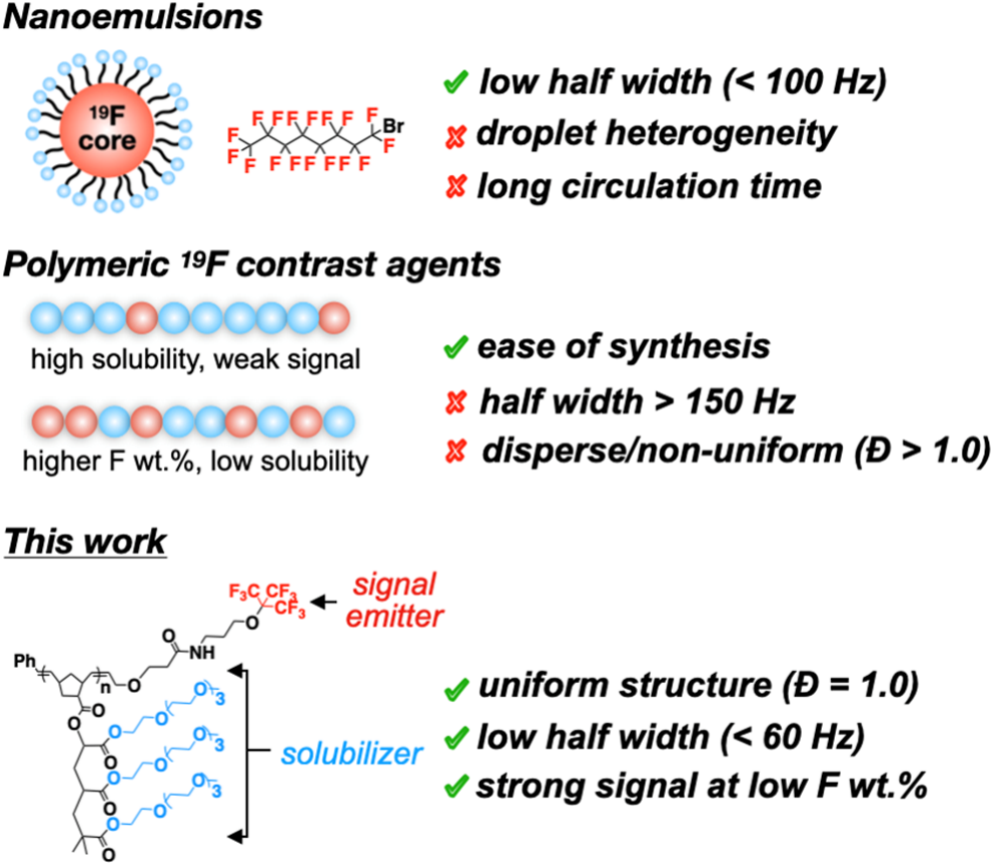
Design Evolution of ^19^F MRI Contrast Agents: Heterogeneous Nano Emulsions, Statistical Semifluorinated Polymers, and Discrete Polymers with an Optimal Balance of Aqueous Solubility and Signal Strength

Among emerging alternatives to standard gadolinium-based MRI contrast agents (CA),^17−23^^19^F MRI CAs present unique opportunities to study structure–property relationships due to their sensitivity to the local environment.^24^ The ^19^F nucleus provides inherent advantages—no background signal, 100% natural abundance, and ^1^H-comparable sensitivity—yet optimal performance demands precise control over fluorine mobility and dynamics. However, current synthetic approaches using statistical copolymerization or postmodification yield heterogeneous products, obscuring correlations between molecular structure and imaging performance. Therefore, semifluorinated polymer-based MRI CAs present a fundamental design challenge: achieving the ideal balance of aqueous solubility and fluorine mobility while preventing signal-diminishing aggregation (Scheme 1).

Previous studies of polymeric ^19^F contrast agents have revealed how molecular design influences signal optimization/quality, with most approaches seeking to maximize fluorine content while maintaining sufficient hydrophilicity for aqueous solubility. The ^19^FIT dendrimer reported by Yu and co-workers,^21^ combining three −C_4_F_9_ groups with four tetraethylene glycol units, demonstrated how precise architectural control achieves both aqueous solubility and strong signal intensity. This molecularly uniform structure remains nonaggregating even above its critical micelle concentration,^25^ highlighting the importance of architectural control in preventing fluorine aggregation. Studies using ROMP-based materials further established how backbone rigidity and fluorine content influence solubility and NMR dynamics—polymers containing −CF_3_/–C_4_F_9_ side chains required significant backbone modification for aqueous solubility yet showed diminished performance above 40 kDa due to restricted fluorine mobility from the aggregation of fluorine blocks and reduced *T*_2_ relaxation times.^26^ Notably, higher fluorine content does not necessarily translate to stronger signals—a dendritic PEG-based polymer with 8 wt % fluorine outperformed its 14 wt % counterpart through better hydrophilic-fluorophilic balance,^27^ indicating that optimal performance depends not on maximizing fluorine content, but rather on precise control of fluorine mobility through architectural design.^28,29^

**Figure 1:**
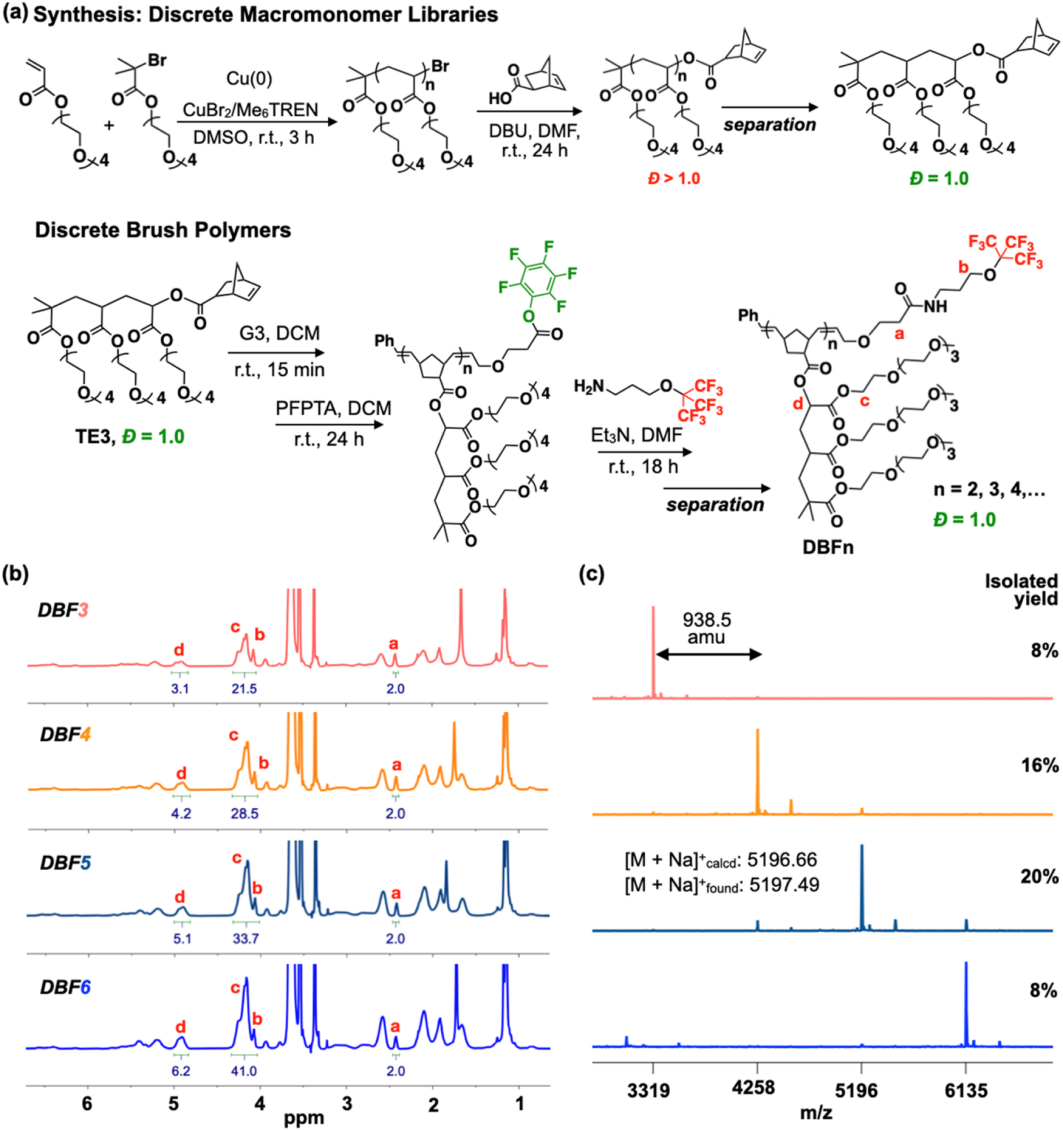
Synthesis and characterization of discrete brush polymers. (a) Synthetic strategy: controlled polymerization followed by chromatographic separation (CLIP) yields discrete macromonomer TE3 (*Đ* = 1.0); subsequent grafting-through ROMP with PFP end-functionalization, postmodification, and separation produces discrete **DBFn** libraries. (b) ^1^H NMR spectra and (c) MALDI-ToF mass spectra confirm the discrete nature of isolated **DBFn** with isolated yields shown for each species.

Here we report the synthesis of discrete brush polymer libraries (*Đ* = 1.0) that enable systematic investigation of how molecular precision influences ^19^F MRI performance. We hypothesize that achieving narrow NMR half-widths through molecular uniformity improves signal quality more effectively than increasing fluorine content. Through controlled polymerization and precise chromatographic separation, we obtain uniform architectures with precise backbone lengths and a single –C_4_F_9_ reporter group placed at the brush end (Figure 1a). This design prioritizes molecular precision and fluorine mobility over fluorine content, incorporating just nine chemically equivalent nuclei at the brush terminus while using discrete oligo(tetraethylene glycol acrylate) side chains for solubility control. Detailed characterization of these discrete species establishes quantitative correlations between molecular architecture and imaging performance, demonstrating capabilities beyond conventional disperse systems.

## Results and Discussion

### Synthesis of Discrete TEG-based Macromonomer

Our approach to prepare discrete brush polymers begins with the synthesis of discrete oligo(tetraethylene glycol) macromonomers (TEG-MM). A tetraethylene glycol bromoisobutyrate initiator (TEGBriB, Figure S2) was first synthesized to maximize hydrophilic content in the final structure. Using this initiator, atom transfer radical polymerization (ATRP) of TEG-acrylate proceeded with excellent control—the linear relationship between ln[*M*_0_]/[*M*_t_] and reaction time confirmed the living nature of the polymerization (first-order kinetics, Figure S22), achieving over 90% monomer conversion within 3 h (oTEG*_n_*-Br, *n* ∼ 3, Figure S3–S4).

Next, we functionalized oTEG-Br with a norbornenyl group through DBU-catalyzed esterification, confirming successful incorporation by the characteristic cyclic alkene protons at 6.2 ppm in the ^1^H NMR spectrum (Figure S5). Sequential purification of this product using flash chromatography (EtOAc/MeOH gradient, Figure S23) followed by recycling size exclusion chromatography (rSEC) enabled the isolation (“CLIPping”) of discrete species in quantitative yield (up to *n* = 5). The molecular uniformity of each macromonomer (TEn) was verified through multiple analytical techniques—quantitative ^1^H NMR integration values and MALDI-ToF mass spectrometry revealed single molecular species (*e.g.*, TE3 [M + Na]^+^ calculated = 961.50, observed = 961.49, Figure S26).

### Synthesis of End-functionalized Discrete Brush Polymers

We synthesized a pentafluorophenyl ROMP terminating agent (PFPTA) following an established procedure (see Supporting Information).^30^ This symmetric internal olefin undergoes deactivating metathesis with living ruthenium centers, introducing PFP end groups that offer distinct advantages over the traditional ethyl vinyl ether termination. The resulting activated ester exhibits high reactivity toward primary amines,^31,32^ providing a versatile synthesis handle for introducing –C_4_F_9_ groups into our brush polymers.

To access discrete fluorinated MRI contrast agents (DBP-TE3_n_-C4F9, termed **DBFn**), we combined grafting-through ROMP with sequential end-group modifications (nucleophilic substitution of PFP) with nonafluoro *t*-butyl groups (−C_4_F_9_), and precision fractionation (Figure 1a). ROMP of discrete TE3 macromonomer yielded a topologically precise brush structure (PBP-TE3_5_-PFP) with an average *N*_BB_ of ∼ 5 (*M_n_* = 4.5 kDa), as confirmed by SEC and NMR analysis. The ^19^F NMR signal at −152 to −162 ppm verified PFP end group incorporation, while integration of the methylene proton (“e”, 4 ppm) in ^1^H NMR confirmed quantitative end-group functionalization (Figure S11). MALDI-ToF analysis revealed a small quantity of α and ω PFP-functionalized species (<10%), presumably due to secondary metathesis of the α (phenyl) chain end with an excess terminating agent during extended reaction times.^33−35^

Conversion from PBP-TE3_5_-PFP to PBP-TE3_5_-C4F9 (**PBF5**) proceeded through PFP-ester chemistry with a synthesized 3-(nonafluoro*-tert*-butoxy)propylamine (see Supporting Information).^30,36,37^ Complete substitution was confirmed by ^19^F NMR, where the PFP signals (−152 to −162 ppm) disappeared entirely while a single new signal appeared at −69 ppm, characteristic of the –C_4_F_9_ moiety (Figure S13). MALDI-ToF analysis further confirmed this transformation through a mass shift of +109 amu, consistent with PFP to –C_4_F_9_ conversion.

Building on our previous work,^11^ we then isolated discrete C_4_F_9_-terminated brush polymer libraries (up to **DBF7**) using recycling preparative SEC, guided by clear identification of individual species in the SEC trace of the parent **PBF5**. This separation yielded discrete products with up to 20% isolated yield for **DBF5**. Comprehensive characterization of these libraries through NMR, SEC, MALDI-ToF, and FTIR analysis (Figures 1, S15–S21, and 30–32) confirmed their discrete nature and purity. The ^1^H NMR spectra showed systematic changes with increasing *N*_BB_ - normalizing to the chain-end methylene peak (“a”, 2.4 ppm), we observed quantitative increases in signals from the TEG methylene peak (“c”, 4.2 ppm) and side chain methine peak(“d”, 4.9 ppm) (Figure 1b). These near-ideal integration values demonstrated the uniform architecture of isolated **DBFn** species.

MALDI-ToF mass spectrometry further confirmed the discrete nature of these materials, showing single peaks separated by 938 amu (the mass of one repeating unit). For **DBF5**, we observed an excellent agreement between calculated and found masses ([M + Na]_calcd_^+^ = 5196.66, and [M + Na]_found_^+^ = 5197.49, Figure 1c). FTIR spectroscopy revealed alternating patterns in the amide stretching region (1660–1700 cm^–1^), where odd-numbered species (*N*_BB_ = 3, 5, 7) show stronger free amide bands (1690 cm^–1^) while even-numbered species (*N*_BB_ = 2, 4, 6) exhibit stronger hydrogen-bonded amide signatures at 1675 cm^–1^ (Figure S32). This systematic variation in –C_4_F_9_ environments correlates with backbone length and manifests in distinct ^19^F NMR behavior, demonstrating direct links between molecular architecture and imaging performance.

### Structure-^19^F NMR Relationships in Discrete Brush Architecture

The clear solution of **PBF5** in PBS/D_2_O (9:1 v/v) demonstrates that discrete TE3 side chains effectively solubilize both the norbornenyl backbone and the terminal –C_4_F_9_ group. At a fluorine concentration of 9.5 mM (5 mg/mL), the ^19^F NMR showed sharp signals (*v*_1/2_ = 57 Hz) and high signal-to-noise ratio (620). Relaxation time measurements of **PBF5** revealed favorable *T*_1_ (spin–lattice, 560 ms) and *T*_2_ (spin–spin, 211 ms) values, while ^19^F DOSY NMR^38^ indicated a hydrodynamic size of 5.5 nm, confirming monomeric behavior in solution (Figure S36). The combination of narrow line width and favorable *T*_2_ relaxation demonstrates effective prevention of fluorine aggregation through our molecular design. However, this system achieves precision only in the side chains—our next goal was to extend this architectural control to the backbone to further enhance NMR performance.

**Figure 2:**
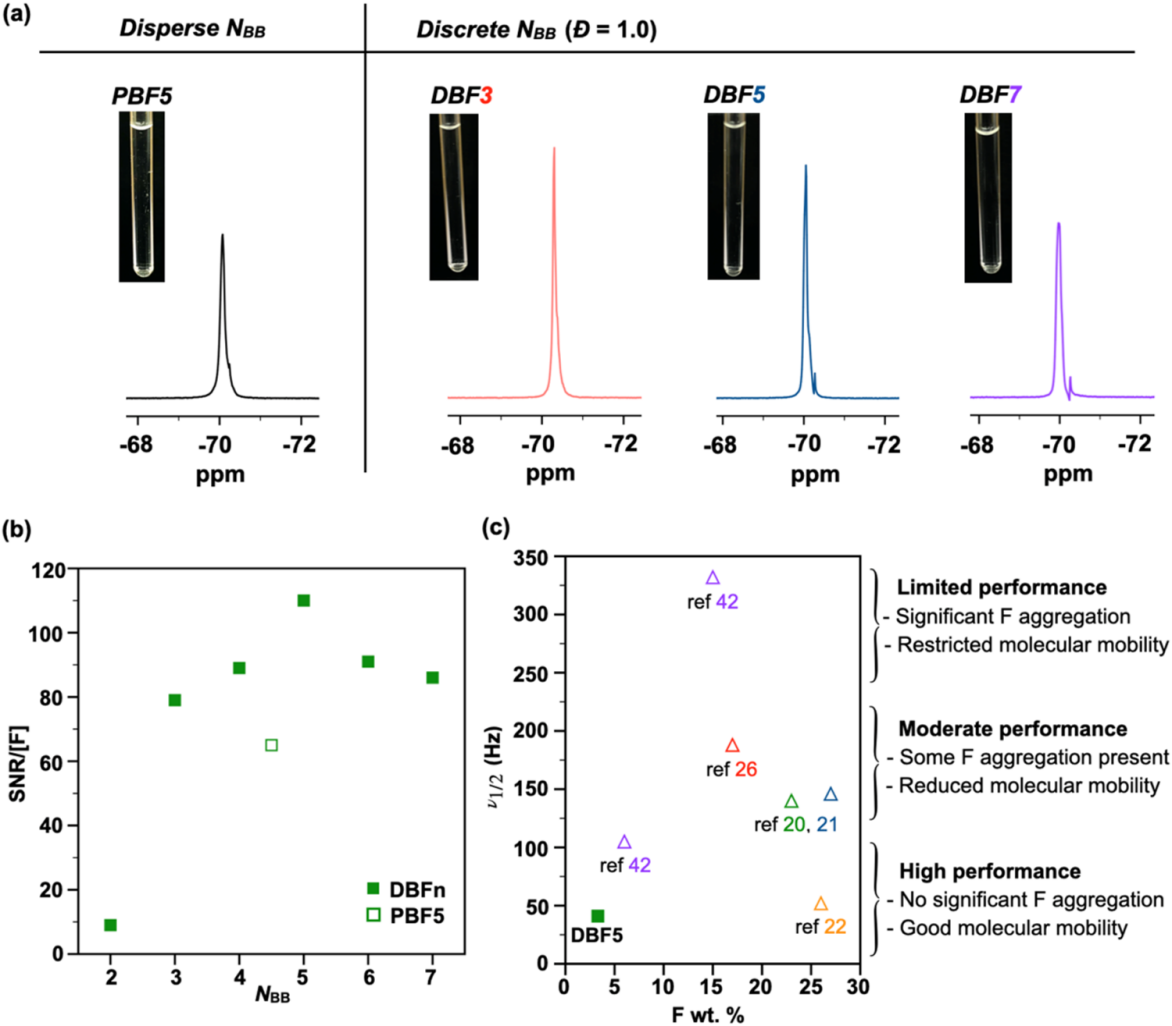
Molecular precision enables high ^19^F NMR performance. (a) ^19^F NMR spectra of disperse backbone **PBF5** and fully discrete **DBFn** libraries in PBS/D_2_O (9:1 v/v, 5 mg/mL), with corresponding solution images showing excellent aqueous solubility. (b) Signal efficiency (SNR/[F]) versus backbone length shows optimal performance at *N*_BB_ = 5, demonstrating superior performance of discrete architectures compared to disperse analogs. (c) Line width analysis versus fluorine content reveals how our brush design achieves sharp signals (41 Hz) despite low fluorine content (3.3 wt %), outperforming conventional high-fluorine materials by preventing fluorine aggregation.

Our investigation of backbone length effects on ^19^F NMR performance revealed systematic trends in aqueous solutions (5 mg/mL) of discrete **DBFn** (*n* = 2 to 7). By normalizing SNR values against fluorine concentration, we found that **DBF5** achieves optimal efficiency with an SNR/[F] value of 110 (Figure 2b). This peak in performance at *N*_BB_ = 5 suggests an ideal balance between aqueous solubility and fluorine mobility. The importance of architectural precision becomes evident when comparing the discrete **DBF5** to its disperse counterpart—both absolute SNR and normalized SNR/[F] values consistently exceed those of **PBF5** (Table 1). This observation reveals a fundamental design principle: preventing fluorine aggregation through molecular architecture proves more effective than maximizing fluorine content. Our design achieves this through two mechanisms: the single –C_4_F_9_ group eliminates intramolecular fluorine clustering, while the brush structure sterically hinders intermolecular aggregation.

**Table 1:** ^19^F Brush Polymer-Based Contrast Agents Investigated in This Study

sample^a^	*Đ*^b^	*M_n_*^b^ (kDa)	*D*_h_ (nm)	F wt %	[F] (mM)	NMR SNR	SNR/[F]	*ν*_1/2_ (Hz)	LCST (°C)
PBF5	1.07^c^	4.7^c^	5.5	3.6	9.5	620	65	57	39
DBF2	1.00	2.4	7.4^d^	7.2	19	175	9	43	26
DBF3	1.00	3.3	4.1^d^	5.2	13.6	1080	79	32	33
DBF4	1.00	4.2	5.9	4.0	10.6	940	89	42	37
DBF5	1.00	5.2	6.1	3.3	8.7	960	110	41	39
DBF6	1.00	6.1	6.1	2.8	7.4	670	91	56	39
DBF7	1.00	7.1	6.1	2.4	6.4	550	86	68	40

a PB—precision brush with discrete side chain and disperse backbone, DB—discrete brush polymer with discrete side chain and backbone. The number represents the backbone length. All data were collected at a polymer concentration of 5 mg/mL in PBS/D_2_O (9:1, v/v).

b Determined from MALDI-ToF experiment.

c Measured using SEC.

d Measured by DLS, size from volume distribution is reported.

D_h_ was measured using ^19^F DOSY NMR. F wt % was calculated by F wt % = mass of 9 fluorine atoms, 171/Mn × 100%. SNR of ^19^F spectra was estimated using the analysis on Bruker Topspin. LCST was determined using DLS.

The ^19^F NMR chemical shift of **DBFn** provides molecular-level evidence for how backbone architecture influences fluorine environments. As *N*_BB_ increases from 2 to 7, the chemical shift moves downfield from −70.51 to −69.96 ppm, as ^19^F resonances are known to be highly sensitive to their local environment.^24,25,39^ Analysis of these shifts reveals a critical transition point between *N*_BB_ = 4 and 5 (Figure S33): below this point, each addition of backbone repeat unit causes significant chemical shift, while above it, the changes become negligible. This transition at *N*_BB_ = 5 coincides with optimal performance^40^ and indicates that additional TE3 side chains beyond this length will not significantly affect the −C_4_F_9_ environment. These chemical shift data directly support our design principle—the structural precision of the backbone can affect fluorine environments and improve ^19^F NMR signal quality.

SNR analysis across the **DBFn** series reveals how backbone length affects signal quality. With the exception of **DBF2**, which shows reduced SNR (175) due to aggregation at the measurement temperature (near its LCST, see below), we observe a systematic trend in performance. As expected, the SNR values decrease from 1080 to 550 as *N*_BB_ increases from 3 to 7, reflecting the lower fluorine content at constant weight concentration (5 mg/mL). Specifically, *N*_BB_ = 3 to 7 show SNR values that decrease systematically from 1080 to 550, corresponding to fluorine content reduction from ∼5 to ∼2 wt % (Table 1 and Figure S34a). The case of **DBF2** underscores a critical design requirement: adequate aqueous solubility must precede optimization of fluorine content. Indeed, lower fluorine content often enables better performance by ensuring molecular dissolution and preventing aggregation.^27^

To understand molecular mobility in these systems, we measured *T*_1_ (spin–lattice) and *T*_2_ (spin–spin) relaxation times at 11.7 T. All samples exhibit relatively short *T*_1_ values (∼550 ms), advantageous for rapid signal acquisition *in vivo*, with minimal dependence on backbone length. Specifically, **DBFn** shows *T*_1_ values of 545, 546, and 550 ms for *N*_BB_ = 3, 5, and 7, respectively (Table S1). The parent material **PBF5** exhibits a slightly longer *T*_1_ (560 ms), suggesting the importance of architectural precision for decreasing relaxation time. For *T*_2_ relaxation, which reflects the segmental mobility of fluorine groups, we observe a systematic improvement with increasing backbone length—from 160 to 210 to 222 ms as *N*_BB_ increases from 3 to 7 (a longer *T*_2_ is desirable for improved imaging, Table S1). This trend supports another design principle: lower fluorine content in longer structures reduces the probability of aggregation,^41^ known to restrict molecular motion and shorten *T*_2_.^42,43^ Notably, **DBF5** and **PBF5** show similar *T*_2_ values (∼210 ms), indicating that backbone dispersity primarily influences *T*_1_ processes while maintaining favorable fluorine mobility. These relaxation parameters, combined with our sharp line widths, translate directly to high-quality MR images.

**Figure 3:**
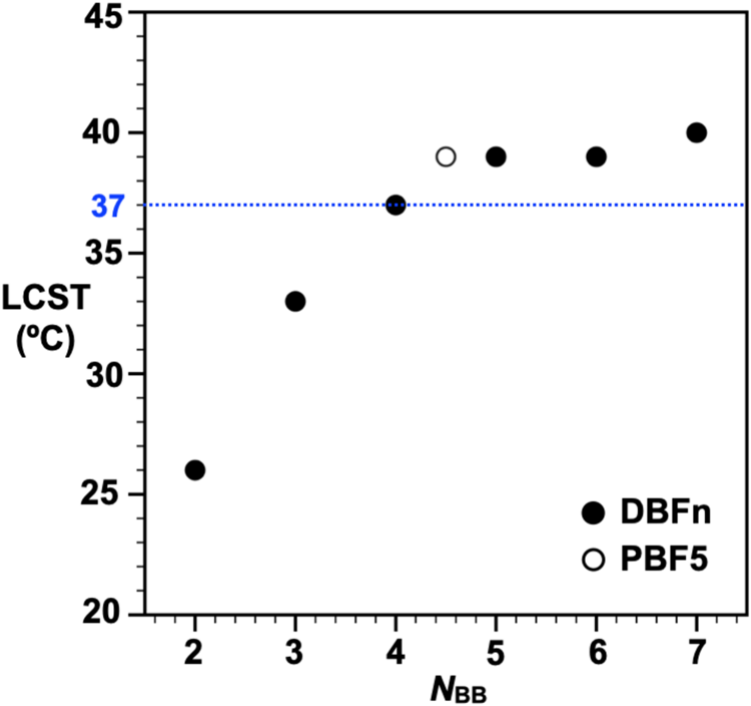
LCST behavior reveals systematic control through backbone architecture. LCST values of discrete DBFn in PBS/D_2_O solution (5 mg/mL) increase with backbone length (*N*_BB_ = 2 to 7) until plateauing above *N*_BB_ = 5. The disperse analog PBF5 (open circle) shows comparable behavior to its discrete counterparts. The dotted line indicates body temperature, highlighting the biological relevance of these transitions.

As mentioned above, the thermoresponsive behavior of **DBFn** exhibits systematic dependence on backbone architecture. Dynamic light scattering shows distinct transitions in hydrodynamic diameter (*D*_h_) as solutions approach their LCST (Figure S40a). At 5 mg/mL, LCST values increase with backbone length, from 26 °C for **DBF2** to 40 °C for **DBF7** (Figure 3), consistent with the established behavior of ethylene glycol-based polymers.^44−47^ The transitions also show a concentration dependence^48^ - **DBF6** exhibits an LCST increase from 39 to 45 °C when concentration decreases from 5 to 2.5 mg/mL (Figure S40c), a feature particularly relevant near body temperature (37 °C). At elevated temperatures, all samples undergo a second transition, forming large aggregates (>10 μm) through a liquid–liquid phase transition. These coacervation temperatures occur between 49–66 °C across the series (*N*_BB_ = 2 to 7), with no clear dependence on backbone length. The PBS salt environment likely promotes this behavior by inducing dehydration, favoring intermolecular associations over polymer–solvent interactions.^49−51^**PBF5** shows similar coacervation at 66 °C but exhibits rapid aggregate collapse at 68 °C, suggesting that molecular dispersity affects coacervation stability.

**Figure 4:**
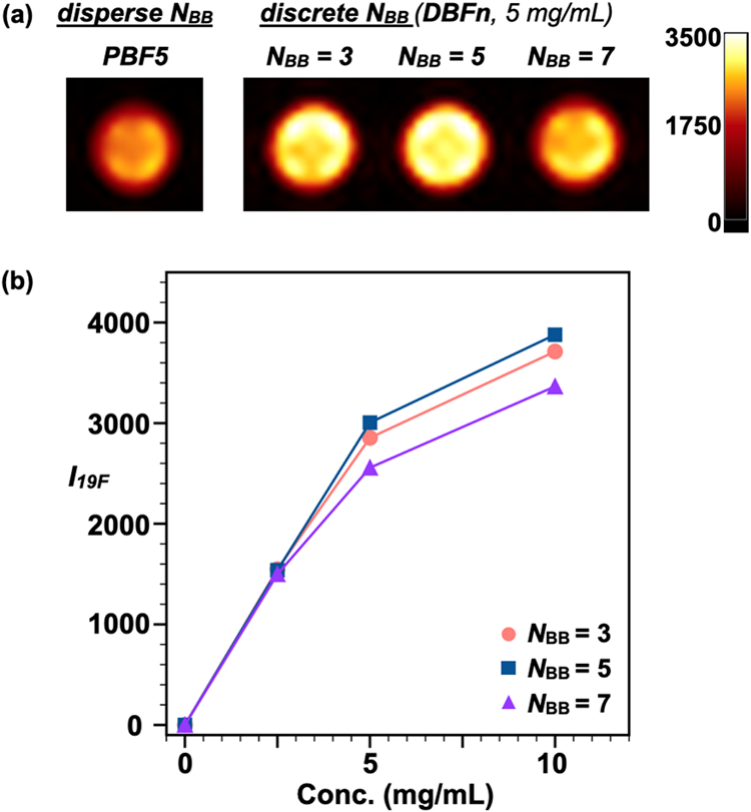
Discrete architecture enables efficient MRI contrast at clinically relevant field strength (3 T) and concentrations. (a) ^19^F MR images of **PBF5** and **DBFn** (*n* = 3, 5, 7) in PBS/D_2_O at 5 mg/mL. (b) Image intensity versus concentration shows high sensitivity down to 2.5 mg/mL, with optimal performance seen for **DBF3** and **DBF5**.

To validate our molecular design at clinically relevant conditions, we performed MRI measurements using a 3 T scanner, comparing discrete DBFn (*n* = 3, 5, 7) with disperse PBF5 in PBS. At 5 mg/mL, DBF3 and DBF5 show the highest ^19^F MRI image intensity (*I*_19F_), while **DBF7** and **PBF5** exhibit weaker signals (Figure 4a). The reduced performance of PBF5 reflects its molecular heterogeneity, where longer chains with lower fluorine content diminish overall signal intensity. Signal strength increases with concentration for all samples but deviates from linearity at 10 mg/mL (Figure 4b), likely due to concentration-dependent LCST effects leading to increased aggregation of fluorine nuclei. Notably, all materials produce detectable signals even at 2.5 mg/mL—a significant improvement over previous reports that typically require higher concentrations. These results demonstrate that precise molecular architecture enables effective imaging at low concentrations even with lower field strengths, potentially reducing the amount of contrast agent needed for imaging applications and toxicity risks.

**Figure 5:**
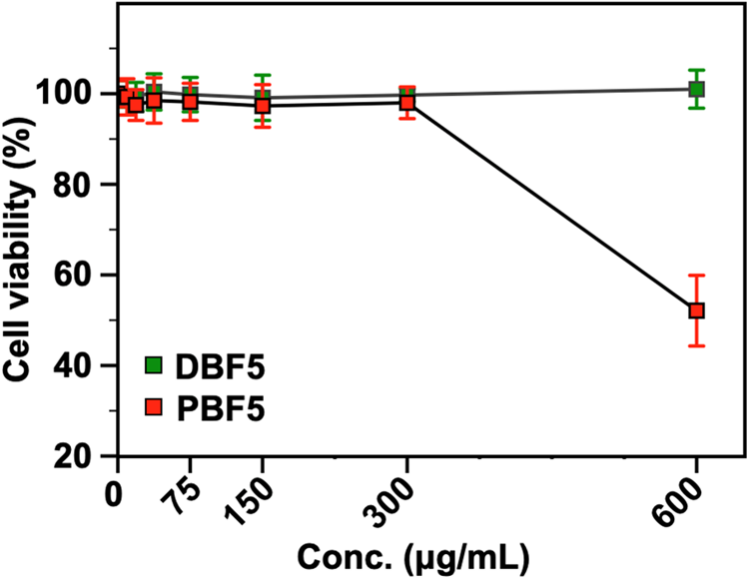
Comparison of cell viability between discrete (**DBF5**) and disperse (**PBF5**) architectures. A549 cells were exposed to increasing polymer concentrations (0–600 μg/mL) for 48 h, showing a superior noncytotoxic nature of the discrete brush polymer sample at high concentrations. Error bars represent the standard deviation from five independent experiments.

The biological compatibility of these contrast agents determines their practical utility. We evaluated cytotoxicity by exposing A549 lung cancer cells to the disperse **PBF5** and discrete **DBF5** at concentrations ranging from 6.25 to 600 μg/mL for 48 h at 37 °C and 5% CO_2_. Cell viability measurements using CellTiter-Glo (CTG) assay^52^ with a serum-free medium control reveal a clear distinction between disperse and discrete architectures at high concentrations. **DBF5** maintains complete cell viability (>99%) across all tested concentrations, while **PBF5** shows significant toxicity above 300 μg/mL, dropping to ∼50% viability at 600 μg/mL (Figure 5). This high noncytotoxicity of the discrete architecture, combined with its efficient imaging performance at low concentrations (2.5–5 mg/mL), suggests DBF5 as a promising candidate for clinical studies. The superior biological tolerance of **DBF5** stems from its uniform structure, which prevents unexpected cellular interactions. In contrast, **PBF5** contains a distribution of lower molecular weight species with LCST values ≤37 °C (the culture temperature), leading to partial fluorous aggregation and undesirable cell interactions at higher concentrations. Since **DBF5** has an LCST > 37 °C, it remains fully soluble during cell culture. To support this hypothesis, we exposed **DBF3** (LCST = 33 °C) to the same cell line, which resulted in significantly reduced cell viability (Figure S46).

## Conclusions

We report discrete brush polymers that establish new design principles for ^19^F MRI contrast agents. While conventional approaches rely on maximizing fluorine content (up to 20 wt %), architectural precision enables superior performance with minimal fluorine incorporation. Through controlled synthesis and chromatographic separation, we access uniform structures with discrete backbone lengths and a single –C_4_F_9_ group (<7 wt %). This design prevents fluorine aggregation through two mechanisms: the isolated –C_4_F_9_ terminus eliminates intramolecular clustering, while the branched architecture and high aqueous solubility prevent intermolecular association. Our scalable synthesis strategy yields discrete architectures that eliminate batch-to-batch variability, ensuring reproducible properties. These materials demonstrate exceptional performance—sharp line width, high SNR, favorable relaxation times, and strong MR contrast at 3T. Systematic variation of backbone length reveals optimal performance at *N*_BB_ = 5, achieving efficient imaging even at low concentrations (2.5 mg/mL) while maintaining excellent noncytotoxicity. This work establishes that molecular precision, rather than fluorine content, determines contrast agent effectiveness—a principle that transforms the design of polymer-based imaging materials.

## Supporting Information Available

Experimental procedures and characterization data (NMR, MALDI-ToF, SEC, FTIR, DLS) for all samples (PDF)
