# Supplementary material for: Discrete Brush Polymers Enhance 19F MRI Performance through Architectural Precision
Source: J Am Chem Soc. 2025 May 1;147(19):16171–8. doi: 10.1021/jacs.5c00938 (PMC12082627; doi:10.1021/jacs.5c00938)
Supplement: Supplementary file 1 — ja5c00938_si_001.pdf [file ja5c00938_si_001.pdf]

Discrete Brush Polymers Enhance 19F MRI Performance through Architectural Precision

Nduka D. Ogbonna,[1] Parikshit Guragain,[1] Venkatesh Mayandi,[2] Cyrus Sadrinia,[3] Raman Danrad,[3] Seetharama Jois,[2] Jimmy Lawrence[1]*

[1]Department of Chemical Engineering, Louisiana State University, Baton Rouge, 70803, United States.

[2]Department of Pathological Sciences, School of Veterinary Medicine, Louisiana State University, Baton Rouge, 70803, United States.

[3]Department of Radiology, School of Medicine, Louisiana State University Health, New Orleans, 70112, United States.

Supporting Information

Table of contents

EXPERIMENTAL PROCEDURES......3

Materials......3

Instrumentation......3

Methods......6

Synthesis of tetraethylene glycol acrylate monomer (TEG-Ac)......6

Synthesis of tetraethylene glycol bromoisobutyrate initiator (TEGBriB)......6

Synthesis of oligo(tetraethylene glycol) (TEG-Br) using Cu(0)-RDRP (target DP = 3)......7

Synthesis of oligo(tetraethylene glycol) macromonomer (NB-TEG) via esterification......7

Synthesis of pentafluorophenyl ROMP terminating agent (PFPTA)......8

Synthesis of 3-amino-1-(nonafluoro-tert-butyoxy)propane hydrochloride......10

Synthesis of PFP-terminated bottlebrush polymer......11

Synthesis of C_4F_9-terminated bottlebrush polymer......11

Figure S1. ^{1}H NMR (400 MHz, $CDCl_3$) and ^{13}C NMR (125 MHz, $CDCl_3$) of tetraethylene glycol acrylate monomer (TEG-Ac)......13

Figure S2. ^{1}H NMR (400 MHz, $CDCl_3$) and ^{13}C NMR (125 MHz, $CDCl_3$) of tetraethylene glycol bromoisobutyrate initiator (TEGBriB)......14

Figure S3. ^{1}H NMR (400 MHz, $CDCl_3$) of a few aliquots collected at intervals for ATRP of TEG-Ac with TEGBriB initiator......15

Figure S4. ^{1}H NMR (400 MHz, $CDCl_3$) of disperse ω-bromo oligo(tetraethylene glycol) $oTEG_3$-Br......16

Figure S5. ^{1}H NMR (400 MHz, $CDCl_3$) and ^{13}C NMR (125 MHz, $CDCl_3$) of discrete ω-norbornenyl oligo(tetraethylene glycol) NB-$oTEG_3$ (TE3) isolated from disperse NB-$oTEG_3$......17

Figure S6. ^{1}H NMR (400 MHz, $CDCl_3$) of di-tert-butyl 3,3'-(but-2-ene-1,4-diylbis(oxy))(Z)-dipropionate 17

Figure S7. ^{1}H NMR (400 MHz, $CDCl_3$) of di-carboxylic acid 3,3'-(but-2-ene-1,4-diylbis(oxy))(Z)-dipropionate......18

Figure S8. ^{19}F NMR (470 MHz), ^{1}H NMR (400 MHz), and ^{13}C NMR (125 MHz) of di-pentafluorophenyl 3,3'-(but-2-ene-1,4-diylbis(oxy))(Z)-dipropionate ROMP terminating agent (PFPTA) in $CDCl_3$......18

Figure S9. ^{19}F NMR (470 MHz), ^{1}H NMR (400 MHz), and ^{13}C NMR (125 MHz) of N-(tert-butyloxycarbonyl)-3-amino-1-(nonafluoro-tert-butoxy)-propane in $CDCl_3$......19

Figure S10. ^{19}F NMR (470 MHz), ^{1}H NMR (400 MHz), and ^{13}C NMR (125 MHz) of 3-amino-1-(nonafluoro-tert-butyoxy)propane hydrochloride in DMSO-d_6......20

Figure S11. ^{19}F NMR (470 MHz) and ^{1}H NMR (500 MHz) of PFP-terminated precision bottlebrush

polymer, PBP-$TE3_5$-PFP in $CDCl_3$ 21
Figure S12. ^1H NMR (500 MHz, $CDCl_3$) of C_4F_9-terminated precision bottlebrush polymer, PBF5 22
Figure S13. ^{19}F NMR (470 MHz, $CDCl_3$) spectra showing the conversion of PBP-$TE3_5$-PFP (top) to PBF5 (bottom) 22
Figure S14. ^{19}F NMR (470 MHz) spectrum of 5 mg/mL PBF5 in PBS/D_2O (9/1 v/v) 23
Figure S15. ^{19}F NMR (470 MHz) and ^1H NMR (500 MHz) of C_4F_9-terminated discrete bottlebrush polymer, DBF2 in $CDCl_3$ 24
Figure S16. ^{19}F NMR (470 MHz) and ^1H NMR (500 MHz) of C_4F_9-terminated discrete bottlebrush polymer, DBF3 in $CDCl_3$ 25
Figure S17. ^{19}F NMR (470 MHz) and ^1H NMR (500 MHz) of C_4F_9-terminated discrete bottlebrush polymer, DBF4 in $CDCl_3$ 26
Figure S18. ^{19}F NMR (470 MHz) and ^1H NMR (500 MHz) of C_4F_9-terminated discrete bottlebrush polymer, DBF5 in $CDCl_3$ 27
Figure S19. ^{13}C NMR (125 MHz) spectrum of C_4F_9-terminated discrete bottlebrush polymer, DBF5 in CDCl3 28
Figure S20. ^{19}F NMR (470 MHz) and ^1H NMR (500 MHz) of C_4F_9-terminated discrete bottlebrush polymer, DBF6 in CDCl3 29
Figure S21. ^{19}F NMR (470 MHz) and ^1H NMR (500 MHz) of C_4F_9-terminated discrete bottlebrush polymer, DBF7 in CDCl3 30
Figure S22. (a) Conversion of TEG-Ac monomer over time (b) $In[M]_0/[M]_t$ vs polymerization time for ATRP of TEG-Ac using TEGBriB 30
Figure S23. SEC profile of disperse oTEG3-Br 31
Figure S24. Gradient flash chromatography profile showing separation of disperse NB-oTEG3 into discrete macromonomer libraries. TE# represents discrete NB-oTEG# and the mass yield of each discrete fraction is included 31
Figure S25. High resolution isolation of discrete NB-oTEG3 (TE3) using rSEC 32
Figure S26. (a) MALDI-ToF spectrum (b) SEC profile of discrete NB-oTEG3 (TE3) 32
Figure S27. SEC profile of PFP-terminated precision bottlebrush polymer PBP-TE35-PFP (blue) from TE3 macromonomer (black) 33
Figure S28. SEC profile showing the conversion of PBP-TE35-PFP (black) to PBF5 (blue) 33
Figure S29. rSEC profile showing the isolation of DBF2-4 from one batch of PBF5 gotten from flash chromatography 34
Figure S30. SEC profile of isolated discrete DBFn libraries 34
Figure S31. MALDI-ToF spectra of isolated discrete DBFn libraries 35
Figure S32. FTIR spectra of isolated discrete DBFn libraries 35
Figure S33. Plot of ^{19}F NMR chemical shift vs N_{BB} for discrete DBFn libraries 36
Figure S34. Plot of ^{19}F NMR (a) SNR and (b) chemical shift vs NBB for discrete DBFn (filled shape) and PBF5 (open shape) libraries 36
Figure S35. Example determination of ^{19}F NMR SNR of PBF5 using Bruker Topspin software 37
Figure S36. Example 19F DOSY NMR fitting of PBF5 and determination of size (Dh) 37
Figure S37. DLS volume distribution trace of discrete DBFn libraries in PBS/D_2O (5 mg/mL, 25 ºC) 38
Figure S38. Plot of size vs NBB of DBFn (filled shape) and PBF5 (open shape) libraries. Data from 19F DOSY NMR is in black, and DLS is in blue. Volume distribution data is reported for DLS 38
Figure S39. Plot of size vs NBB of DBFn (filled shape) and PBF5 (open shape) libraries at 25 ºC (black triangle) and 37 ºC (blue circle). Data from DLS volume distribution 39
Figure S40. Size vs temperature trend of (a) DBFn and PBF5 libraries in PBS/D2O (9/1 v/v) (b) DBF5 in D2O, 2nd aggregation into >10 microns only observed in PBS/D_2O. (c) DBF6 in PBS/D_2O at 5 and 2.5 mg/mL 40

Figure S41. Example ^{19}F NMR T_1 relaxation fitting of DBF5 40
Figure S42. Example ^{19}F NMR T_2 relaxation fitting of DBF5 41
Figure S43. CTG assay on different concentrations of DBF5 42
Figure S44. CTG assay on different concentrations of PBF5 44
Figure S45. CTG assay on different concentrations of DBF3 45
Figure S46. Comparison of cell viability of DBF5, DBF3, and PBF5. A549 cells were exposed to increasing concentrations (0-600 μg/mL) for 48 h 46

EXPERIMENTAL PROCEDURES

Materials

All reagents were purchased from Sigma-Aldrich/Millipore and used without further purification unless otherwise specified. Copper (II) bromide and 2-Bromo-2-methylpropionyl bromide (98%) were purchased from Acros Organics. Pentafluorophenol (99%) was obtained from BeanTown Chemical, while *cis*-2-butene-1,4-diol (97%) was purchased from Thermo Scientific. Tetraethylene glycol monomethyl ether (>98%) and nonafluoro-*tert*-butyl alcohol (>98%) were purchased from TCI chemicals. Solvents for chromatographic processes were purchased from VWR chemicals and used as received. Deuterated solvents were purchased from Cambridge Isotope Laboratories.

Instrumentation

All reactions were conducted in oven-dried glassware under an inert atmosphere. Reaction progress was monitored via analytical thin-layer chromatography (TLC) using silica gel 60G F_{254} TLC plates (EMD Millipore), and visualized using 254 nm UV, bromocresol green, or potassium permanganate. Sample purification was carried out using automated flash chromatography on a Biotage Isolera One unit equipped with an evaporative light scattering detector (ELSD, Teledyne ISCO). Purifications utilized Biotage KP-SIL SNAP/SNAP Ultra cartridges (25/50/100/340 g) or Silicycle cartridges with the appropriate solvent gradient. High-resolution polymer purifications and the isolation of discrete libraries were achieved through preparative-scale recycling size exclusion chromatography (rSEC) using a LaboACE LC-5060 system equipped with a cross-linked polystyrene/divinylbenzene (PS/DVB) column (JAIGEL series), eluting with chloroform.

Gel permeation chromatography (GPC) was performed on a TOSOH HLC-8320GPC equipped with a TSKgel superH5000 column (3 µm particle and 20 nm pore size), eluting with tetrahydrofuran (THF). Absolute molecular weight analysis was conducted with a Wyatt Dawn EOS multi-angle light scattering (MALS) detector (λ = 658 nm) and processed using Astra 6 software. Weight-averaged molecular weights (M_w) and number-averaged molecular weights (M_n) were calculated relative to linear polystyrene or in-house discrete bottlebrush standards unless otherwise stated.

^{1}H, ^{13}C, and ^{19}F Nuclear magnetic resonance (NMR) spectra were recorded on Bruker Avance III 400 or 500 MHz spectrometers at 298 K. Chemical shifts (δ) are reported in parts per million (ppm) and referenced to the deuterated solvent signal. ^{19}F NMR spectra of samples in PBS/D_2O (9/1, v/v) were recorded on a Bruker Avance III 500 MHz spectrometer at 298 K. Solution spectra were measured with the following parameters: 90º pulse width of 11 µs, relaxation delay of 1 s, acquisition time of 0.58 s, and 64 scans. Diffusion-ordered (DOSY) spectra were obtained on a Bruker Avance III 500 MHz spectrometer at 298 K using the standard ledbpgp2s pulse sequence. The diffusion delay (d20), diffusion gradient length (p30), and the number of gradient steps were set to 170 ms, 1.5 ms, and 16, respectively. A relaxation delay of 3 s and 16 scans were used. Topspin 3.2 software was used to obtain ^{19}F SNR and to process the DOSY spectra.

Spin-Lattice relaxation times (T_1) were measured using the inversion-recovery pulse sequence pulse sequence on a Bruker Avance III 500 MHz spectrometer at 298 K. The samples were dissolved in PBS/D_2O (9/1, v/v) at a concentration of 5 mg/mL. For each measurement, 16 scans, 6 s relaxation delay, 11 µs pulse width was used, and 16 slices were collected with inverse recovery delay from 0.05 to 10 s. The data was fit using the invrec function, allowing the calculation of T_1. Spin-Spin relaxation times (T_2) were measured using the standard Carr-Purcell-Meiboom-Gill (CPMG) sequence on a Bruker Avance III 500 MHz spectrometer at 298 K. The samples were dissolved in PBS/D_2O (9/1, v/v) at a concentration of 5 mg/mL. For each measurement, 16 scans, 5 s relaxation delay was used, and 12 slices were collected with the pulse train lengths from 2 to 200. The decay in the amplitude of the spin echo could be described by an exponential function, allowing the calculation of T_2.

Fourier-transform infrared (FTIR) spectra were recorded on a Bruker Tensor 27 system equipped with a room-temperature DTGS detector, a mid-IR source (4000 - 400 cm^{-1}), and a KBr beam splitter. Samples were analyzed using a Pike Miracle single-bounce attenuated total reflectance (ATR) cell fitted with a ZnSe single crystal.

The spectral resolution was set to 4 cm^{-1}, and 32 scans were collected for each sample. Data processing was performed using Bruker OPUS software.

Matrix-assisted laser desorption/ionization time-of-flight mass spectrometry (MALDI-ToF-MS) spectra were obtained using a Bruker UltrafleXtreme tandem time-of-flight mass spectrometer in positive reflectron mode. Unless otherwise stated, MALDI spectra were collected using a trans-2-[3-(4-tert-butylphenyl)-2-methyl-2-propenylidene]malononitrile (DCTB)/sodium trifluoroacetate (Na-TFA) matrix mixed with the sample and deposited on a Bruker MTP 384-polished steel target plate. MALDI data were analyzed using FlexAnalysis software.

Dynamic light scattering (DLS) measurements were conducted using a BeNano 180 Zeta Pro analyzer equipped with a 50 mW solid-state laser operating at 671 nm and a detection angle of 173º. The intensity and volume-weighted hydrodynamic diameter were obtained from the analysis of the autocorrelation functions using the method of the cumulants. Aqueous solutions were prepared at a concentration of 5 mg/mL in PBS/D_2O.

^{19}F MR images of polymer solutions were acquired on a 3 Tesla Philips Ingenia MRI. Polymer solutions, loaded in a 10 mm glass vial, were suspended within a foam pad. Turbo Spin Echo (TSE) pulse sequence (TE = 0.97 ms, TR = 2.15 ms, echo train length = 120, 120 x 120 matrix size, 1 (no) averages) was used to obtain T_1-weighted images. A multi-echo gradient-spin-echo (mGraSE) sequence (TE = 17 ms, TR = 800 ms, TE spacing = 8.8 ms, 9 echoes, 1 (no) averages, 120 x 120 matrix size) was used to produce a series of images with different T2 weightings.

Cell viability was evaluated using the CellTiter-Glo® 2.0 Luminescent Cell Viability Assay (Promega #G9242) following the manufacturer's protocol. Briefly, A-549 lung cancer cells (passage 3) were seeded into white, opaque 96-well plates at a density of 10,000 cells per well and allowed to adhere overnight. A comparison of cell viability between discrete (DBF5) and disperse (PBF5) architectures was performed by exposing A-549 lung cancer cells to the disperse PBF5 and discrete DBF5 at concentrations ranging from 6.25 to 600 µg/mL for 48 h at 37 °C and 5% CO_2. Cell viability measurements using the CTG assay included a serum-free medium control. Following treatment, an equal volume of CellTiter-Glo® reagent was added to each well. The plate was gently mixed on a shaker for 2 minutes to facilitate cell lysis and ATP release. After a 10-minute incubation at room temperature, luminescence was measured using a BioTek Synergy H1 microplate reader (BioTek Instruments, Inc., Winooski, VT, USA). Background luminescence from wells containing only medium and reagent was subtracted, and relative cell viability was calculated by normalizing to untreated controls. Each condition was performed in quintuplicate, and results were expressed as mean ± standard deviation.

Methods

Synthesis of tetraethylene glycol acrylate monomer (TEG-Ac)

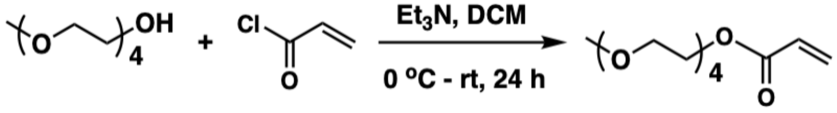

5 g (24 mmol) of tetraethyleneglycol monomethyl ether was taken in an oven-dried RBF with anhydrous DCM (40 mL) and a stir bar. This was cooled to 0 ºC and degassed while stirring for 20 min. Triethylamine (5 mL, 36 mmol) was added to the mixture, followed by a dropwise addition of acryloyl chloride (2.9 mL, 36 mmol) in DCM (10 mL) using an addition funnel. The reaction was allowed to stir overnight, warming to room temperature. After the reaction, DI water was added to the mixture which was extracted with DCM. The combined organic phase was washed with brine and dried over Na_2SO_4. Excess solvent was removed, and the crude was purified by passing through a column (Hex/EtOAc mobile phase). Fractions from purification were combined and characterized. (75% yield).

^{1}H NMR (400 MHz, $CDCl_3$): δ ppm 6.42 (dd, J = 16 Hz, 1H), 6.18 – 6.12 (q, 1H), 5.83 (dd, J = 8.4 Hz, 1H), 4.31 (t, 2H), 3.74 (t, 2H), 3.68 – 3.62 (m, 10H), 3.56 – 3.53 (m, 2H), 3.37 (s, 3H). ^{13}C NMR (125 MHz, $CDCl_3$): δ ppm 166.2, 131.0, 128.3, 71.9, 70.6, 69.1, 63.7, 59.0.

Synthesis of tetraethylene glycol bromoisobutyrate initiator (TEGBriB)

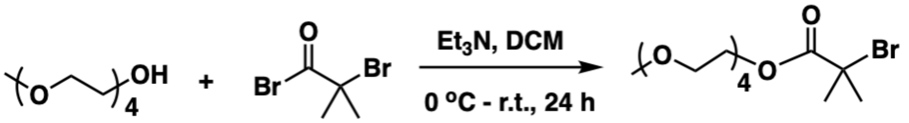

2 g (9.6 mmol) of tetraethyleneglycol monomethyl ether was taken in an oven-dried RBF with anhydrous DCM (13 mL) and a stir bar. This was cooled to 0 ºC and degassed while stirring for 20 min. Triethylamine (2 mL, 14.4 mmol) was added to the mixture, followed by a dropwise addition of 2-Bromo-2-methylpropionyl bromide (1.8 mL, 14.4 mmol) in DCM (5 mL) using an addition funnel. The reaction was allowed to stir overnight, warming to room temperature. After the reaction, DI water was added to the mixture which was extracted with DCM. The combined organic phase was washed with brine and dried over Na_2SO_4. Excess solvent was removed, and the crude was purified by passing through a column (Hex/EtOAc mobile phase). Fractions from purification were combined and characterized. (88% yield).

^{1}H NMR (400 MHz, $CDCl_3$): δ ppm 4.32 (t, 2H), 3.74 (t, 2H), 3.69 – 3.62 (m, 10H), 3.57 – 3.52 (m, 2H), 3.38 (s, 3H), 1.94 (s, 6H). ^{13}C NMR (125 MHz, $CDCl_3$): δ ppm 171.7, 71.9, 70.7, 70.6, 70.5, 68.8, 65.2, 59.1, 55.7, 30.8.

Synthesis of oligo(tetraethylene glycol) (TEG-Br) using Cu(0)-RDRP (target DP = 3)

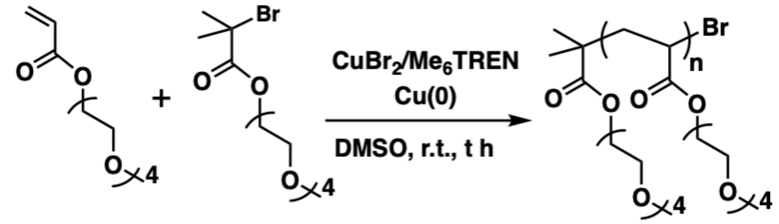

Copper wire (8 cm) was immersed in HCl for 20 min, rinsed with DI water and acetone, and dried. To an oven-dried 40 mL vial, copper(II)bromide ($CuBr_2$) (0.05 equiv.), tris[2-(dimethylamino)ethyl]amine (Me_6TREN) (0.2 equiv.), and dimethylsulfoxide (DMSO, 3 mL) were added, and the contents were sonicated for 10 min. TEG-Ac monomer (3 g, 2.5 equiv.), TEGBriB initiator (1 equiv.), and a stir bar wrapped with the prepared copper wire were added to the vial and degassed with Ar for 20 min. Removing the argon stream, the reaction proceeded at room temperature while stirring until completion. Reaction progress was monitored via NMR analysis of samples taken at predetermined intervals. The polymerization was terminated by quenching in liquid nitrogen, opening to the atmosphere, and diluting with ethyl acetate. The sample was passed through a plug of neutral alumina to remove the catalyst, extracted with DI water, washed with brine, and dried over Na_2SO_4. Excess solvent was evaporated under reduced pressure. (84% yield).

^{1}H NMR (400 MHz, $CDCl_3$) DP ~ 3: δ ppm 4.41 – 4.09 (m, 6H 1H), 3.76 – 3.57 (m, 36H), 3.57 – 3.49 (m, 6H), 3.37 (s, 9H), 2.96 – 1.92 (m, 2H 2H 1H), 1.28 – 1.12 (m, 6H). $M_{n,SEC}$ = 0.7 kDa, $Đ$ = 1.10.

Synthesis of oligo(tetraethylene glycol) macromonomer (NB-TEG) via esterification

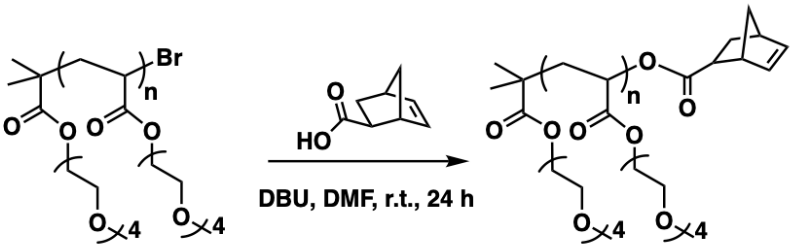

To an oven-dried 40 mL vial containing $oTEG_3$-Br (4000 mg, 5.4 mmol, 1 equiv.), dimethylformamide (DMF, 20 mL) was added to dissolve the sample. Exo-5-Norbornene-2-carboxylic acid (2 equiv) was added to the vial,

followed by dropwise addition of 1,8-diazabicyclo[5.4.0]undec-7-ene (DBU, 2 equiv). The reaction proceeded at room temperature while stirring for 24 h, and reaction progress was monitored via thin-layer chromatography.

DI water (40 mL) and ethyl acetate (60 mL) were added to the reaction mixture in a separatory funnel. After separation into organic and aqueous phases, the organic layer was collected, and the aqueous layer was extracted (3x) with water. The combined organic extract was washed with brine (3x), dried over sodium sulfate (Na_2SO_4), and filtered. Excess solvent was reduced in vacuo to obtain ω-norbornene oligo(tetraethylene glycol) macromonomers. The crude sample was purified by column chromatography eluting with a gradient of EtOAC/MeOH to isolate discrete macromonomer libraries (81% yield for all combined discrete libraries).

Discrete macromonomer libraries were further purified by recycling size exclusion chromatography (rSEC)

Discrete NB-oTEG3 (**TE3**)

^{1}H NMR (400 MHz, $CDCl_3$): δ ppm 6.16 – 6.09 (m, 2H), 5.07 – 4.92 (dm, J = 34.6 Hz, 1H), 4.31 – 4.12 (m, 6H), 3.70 – 3.60 (m, 36H), 3.56 – 3.52 (m, 6H), 3.37 (s, 9H), 3.17 (s, 0.5H), 3.09 (d, J = 7 Hz, 0.5H), 2.92 (s, 1H), 2.67 – 2.55 (m, 1H), 2.31 – 2.25 (m, 1H), 2.24 – 2.05 (m, 2H), 2.01 – 1.91 (m, 2H), 1.76 – 1.66 (m, 1H), 1.54 – 1.48 (m, 1H), 1.44 – 1.31 (m, 2H), 1.21 – 1.12 (m, 6H).
^{13}C NMR (125 MHz, $CDCl_3$) δ ppm 177.0, 175.5, 169.8, 138.2, 135.6, 71.9, 70.6, 69.0, 64.4, 63.7, 59.0, 46.5, 42.6, 41.6, 38.9, 35.3, 34.8, 30.3, 25.8, 24.6. $M_{n,MALDI\text{-}ToF}$ = 0.9 kDa, $Đ$ = 1.00.

Synthesis of pentafluorophenyl ROMP terminating agent (PFPTA)

Step 1 – Michael's addition of tert-butyl acrylate to cis-2-butene-1,4-diol

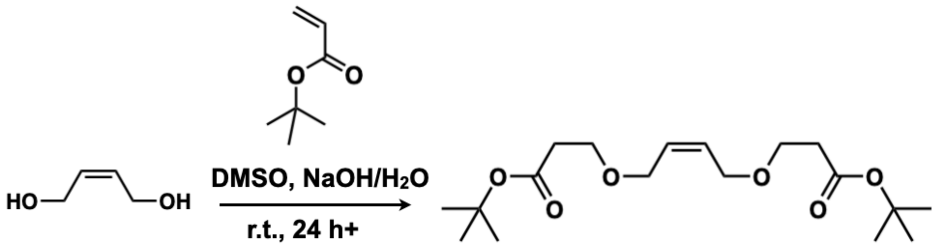

To an oven-dried 40 mL vial containing cis-2-butene-1,4-diol (2 g, 22.7 mmol) in DMSO (30 mL), tert-butyl acrylate (2) (10 mL, 68 mmol, 3 equiv.) was added. A catalytic amount of NaOH (90 mg) in DI water (0.4 mL) was added, and the reaction was stirred at room temperature overnight. After the reaction, 60 mL of water was added, and the mixture was extracted with DCM (3x), the combined organic extract was washed with saturated $NaHCO_3$,

and dried over $MgSO_4$. The excess solvent was removed, and the sample was purified via column chromatography eluting with Hexane/EtOAc (61% yield).

^1H NMR (400 MHz, $CDCl_3$): δ ppm 5.70 (t, 2H), 4.06 (d, J = 4.8 Hz, 4H), 3.66 (t, J = 7.7 Hz, 4H), 2.47 (t, J = 6.9 Hz, 4H), 1.45 (s, 18H).

Step 2 – Hydrolysis of the tert-butyl group

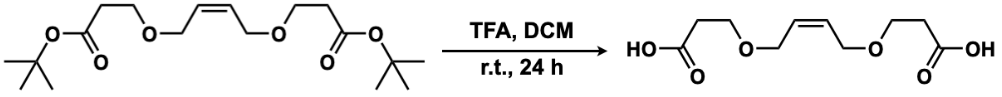

To an oven-dried vial containing di-*tert*-butyl product (4.5 g, 13 mmol) from step 1, excess TFA (10 mL, 10 equiv.) was added, and the mixture was stirred at room temperature overnight. The solvent was removed under reduced pressure. Excess DCM was added and evaporated three times for azeotropic removal of excess acid. The solid dicarboxylic acid product was crystallized from Hexane/EtOAc (4/1) (79% yield).

^1H NMR (400 MHz, $CDCl_3$): δ ppm 9.23 (s, 2H), 5.73 (t, J = 4.8 Hz, 2H), 4.08 (d, J = 4.8 Hz, 4H), 3.72 (t, J = 7.2 Hz, 4H), 2.63 (t, J = 7.2 Hz, 4H).

Step 3 – Esterification of dicarboxylic acid with pentafluorophenol

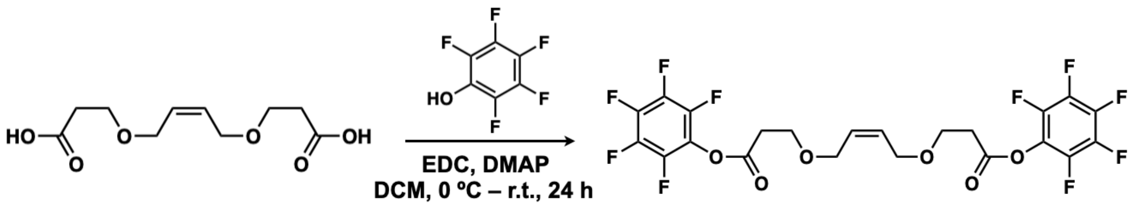

To an oven-dried vial containing dicarboxylic acid product (2000 mg, 8.6 mmol) from step 2 in DCM (15 mL), EDC.HCl (4121 mg, 21.5 mmol, 2.5 equiv.) was added, and the mixture was cooled to 0 ºC. Pentafluorophenol (3900 mg, 21.5 mmol, 2.5 equiv.) and DMAP (210 mg, 1.7 mmol, 0.2 equiv.) in DCM (6 mL) were added under Ar. After stirring for 10 minutes, the reaction mixture was brought to room temperature and stirred for 24 h. After the reaction, DI water was added, and the content was extracted with DCM (3x); the combined organic layers were washed with saturated $NaHCO_3$, and dried over $MgSO_4$. The sample was concentrated and purified by column chromatography eluting with Hexane/EtOAc (87% yield).

^1H NMR (400 MHz, $CDCl_3$): δ ppm 5.75 (t, J = 4.8 Hz, 2H), 4.12 (d, J = 4.4 Hz, 4H), 3.82 (t, J = 4.4 Hz, 4H), 2.93 (t, J = 6.8 Hz, 4H).

^{13}C NMR (125 MHz, $CDCl_3$) δ ppm 167.6, 142.3, 140.2, 139.6, 137.0, 129.4, 67.0, 65.2, 34.6.

^{19}F NMR (376 MHz, $CDCl_3$): δ ppm -152.3 (d, 2F, ortho), -157.6 (t, 1F, para), -162.1 (t, 2F, meta).

Synthesis of 3-Amino-1-(nonafluoro-tert-butyoxy)propane Hydrochloride

Step 1 – Mitsunobu coupling of perfluoro-tert-butanol to N-Boc-protected aminopropan-1-ol

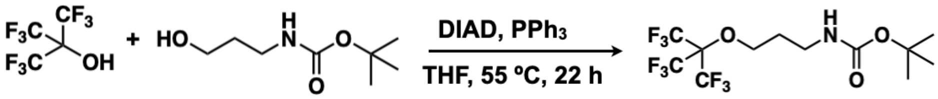

To an oven-dried RBF containing triphenylphosphine (4446 mg, 16.9 mmol), N-Boc-protected aminopropan-1-ol (1980 mg, 11.3 mmol) was added, the flask sealed, and anhydrous THF (20 mL) added under Ar. Next, DIAD (3327 µL, 16.9 mmol) pre-dissolved in 5 mL of THF was added dropwise. Then, perfluoro-*tert*-butanol in 6 mL of THF was added rapidly in one portion at 0 °C. The reaction mixture was brought to 55 ºC and stirred overnight. After the reaction, the crude product was concentrated and purified by flash chromatography eluting with Hexane/EtOAc (95% yield).

^{1}H NMR (500 MHz, $CDCl_3$): δ ppm 4.63 (s, 1H), 4.08 (t, 2H), 3.24 (m, 2H), 1.89 (quint, 2H), 1.43 (s, 9H)

^{19}F NMR (376 MHz, $CDCl_3$): δ ppm -70.14 (s)

Step 2 – Deprotection of *t*-boc group

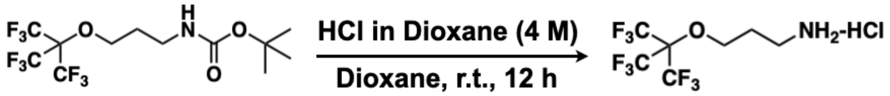

To an oven-dried RBF, HCl in dioxane (14 mL) was added, and the mixture was cooled with an ice bath. N-(*tert*-butoxycarbonyl)-3-amino-1-(nonafluoro-tert-butoxy)-propane (4150 mg, 10.6 mmol) in dioxane (6 mL) was introduced into the flask while stirring under Ar. The mixture was brought to room temperature and stirred overnight. After the reaction was stopped, the crude product was concentrated, the residue was washed with dry ethyl ether, and collected by vacuum filtration as a white solid (82% yield).

^{1}H NMR (400 MHz, DMSO-d_6): δ ppm 8.05 (s, 3H), 4.19 (t, 2H), 2.86 (t, 2H), 1.97 (quint, 2H).

^{13}C NMR (125 MHz, DMSO-d_6) δ ppm 124.8, 121.9, 119.0, 116.1, 79.5, 68.4, 35.7, 27.9.

^{19}F NMR (376 MHz, DMSO-d_6): δ ppm -69.62 (s).

Synthesis of PFP-terminated bottlebrush polymer

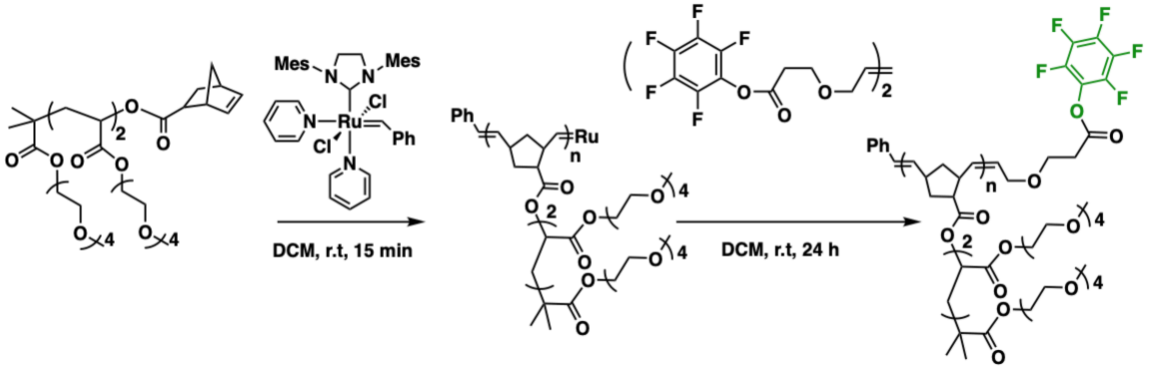

DCM was degassed with Ar for 30 min prior to use.

To an oven-dried vial equipped with a stir bar, **TE3** macromonomer (300 mg, 0.31 mmol, 4.5 equiv.) dissolved in DCM was added and degassed with Ar for 20 min. A degassed DCM solution of Grubbs 3rd generation catalyst $(IMesH_2)(Cl)_2(C_5H_5N)_2Ru{=}CHPh$ (**G3**) (0.07 mmol, 1 equiv.) was injected into the mixture to initiate the polymerization. The mixture was stirred at room temperature for 15 min. PFP ROMP terminating agent (789 mg, 1.4 mmol) in DCM (4.2 mL) was added to the reaction, which was stirred overnight. The crude product was purified by flash chromatography eluting with Hex/EtOAc/MeOH (95% yield).

Sample ID - PBP-$TE3_5$-PFP, $M_{n,SEC}$ = 4.5 kDa, $Đ$ = 1.07.

Synthesis of C4F9-terminated bottlebrush polymer

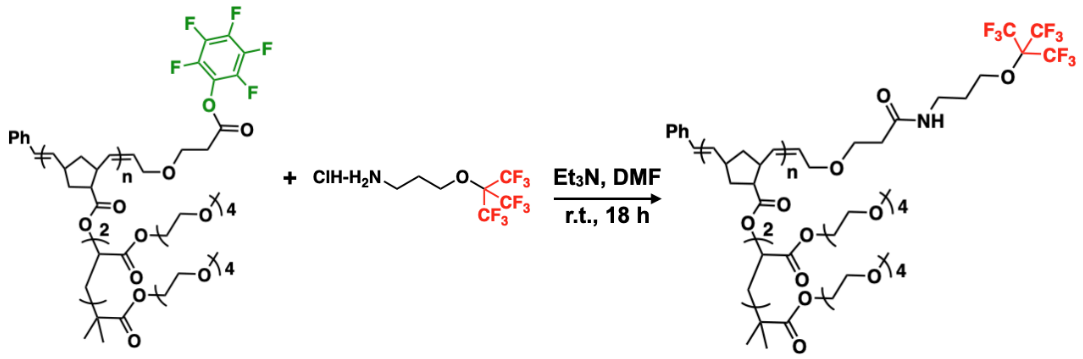

To an oven-dried vial equipped with a stir bar, PBP-$TE3_5$-PFP (268 mg, 0.06 mmol), 3-Amino-1-(nonafluoro-tert-butyoxy)propane Hydrochloride (43 mg, 0.12 mmol), triethylamine (11 µL, 0.08 mmol), and anhydrous DMF (1 mL) were added. The mixture was degassed with Ar for 20 min and was allowed to stir overnight at room temperature. After the reaction, 20 mL of DI water was added and the mixture was extracted

with EtOAc (3x), the combined organic extract was washed with brine, and dried over Na_2SO_4. The crude product was concentrated and purified by flash chromatography eluting with EtOAc/MeOH (86% yield)

Sample ID - PBP-TE3_5-C4F9, $M_{n,SEC}$ = 4.5 kDa, $Đ$ = 1.07.

Discrete bottlebrush libraries DBP-TE3_n-C4F9 were isolated using recycling size exclusion chromatography (rSEC)

Note: DBP-TE3_n-C4F9 and PBP-TE3_5-C4F9 are subsequently referred to as DBFn and PBF5, respectively.

CHARACTERIZATION DATA

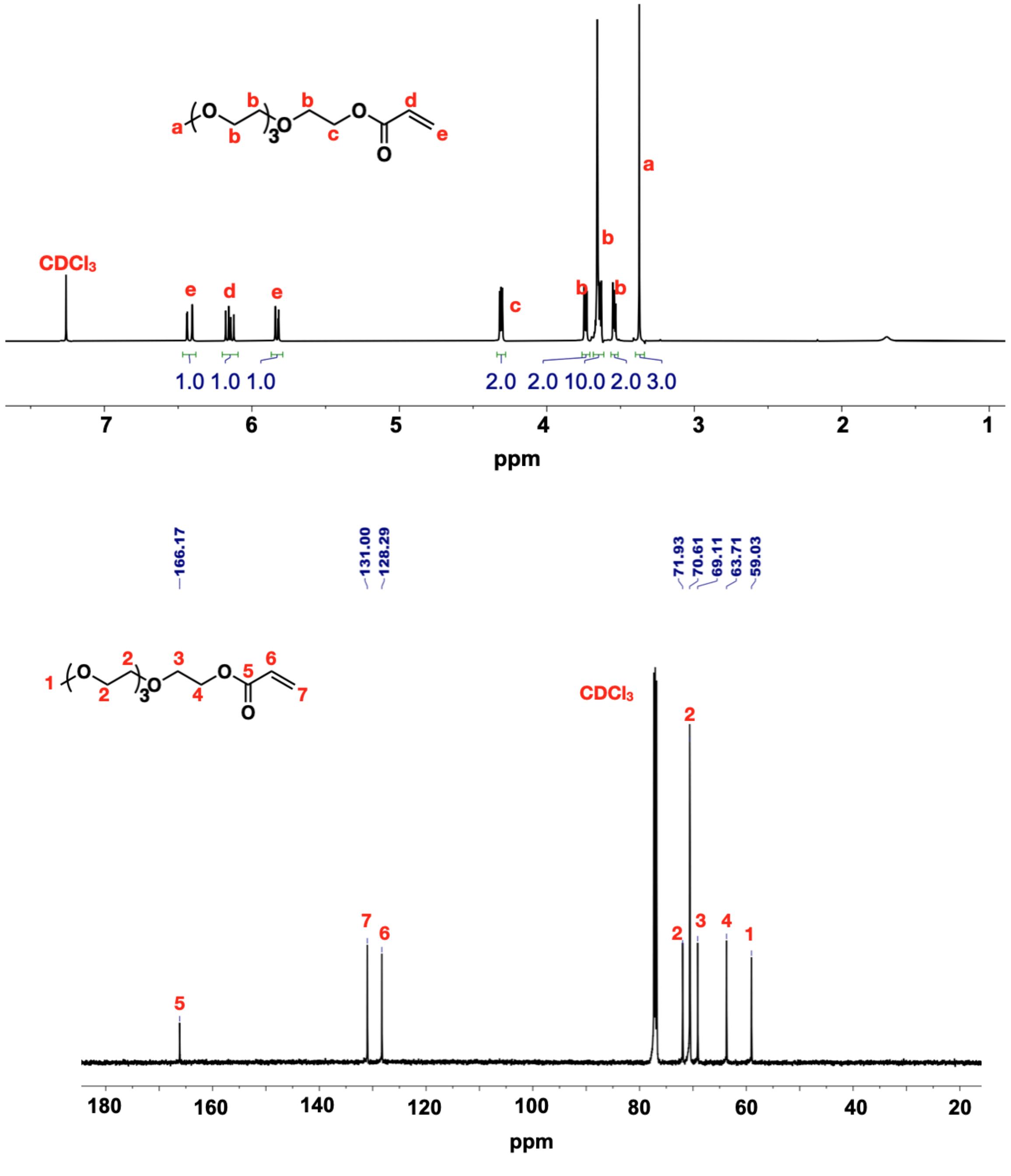

Figure S1. ^{1}H NMR (400 MHz, $CDCl_3$) and ^{13}C NMR (125 MHz, $CDCl_3$) of tetraethylene glycol acrylate monomer (TEG-Ac).

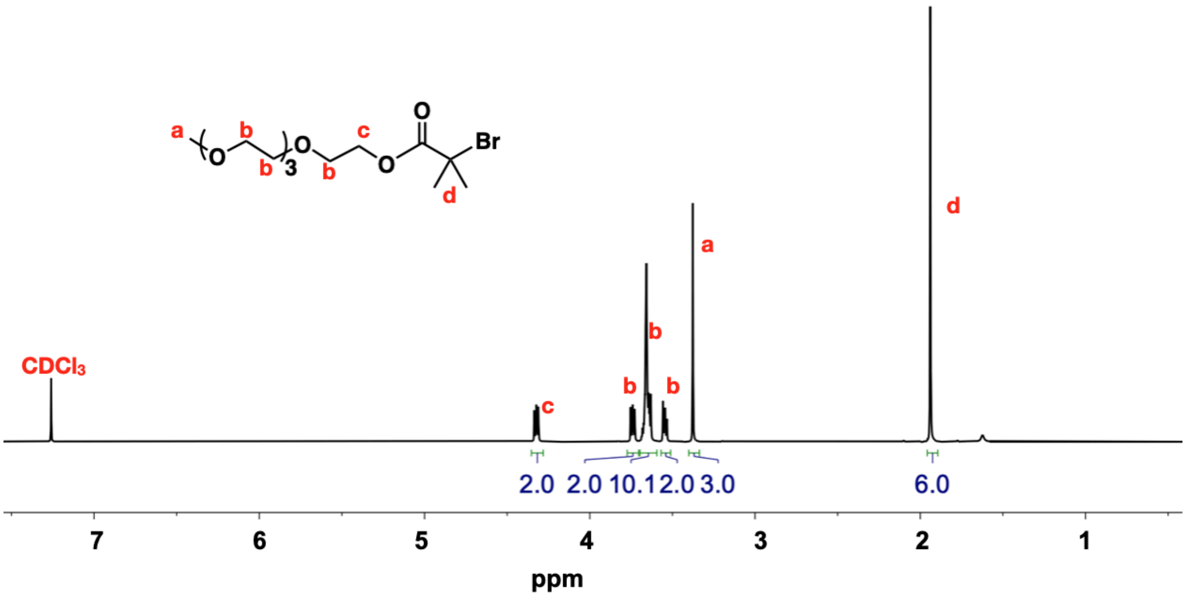

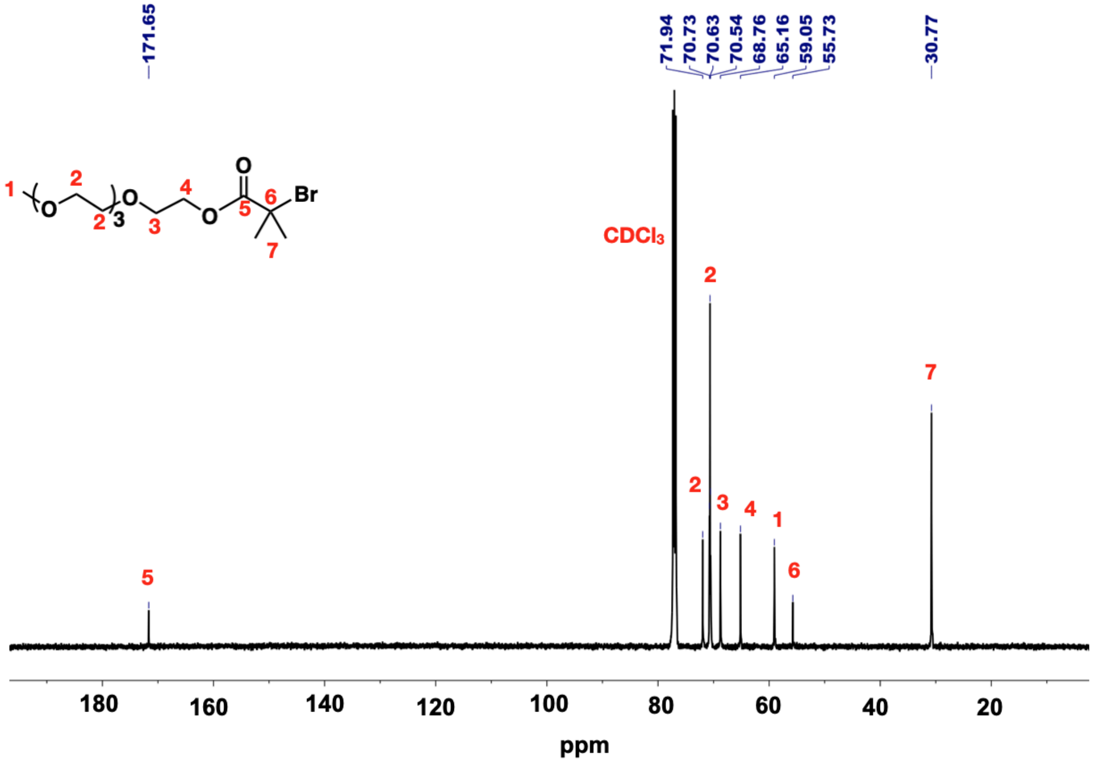

Figure S2. ^{1}H NMR (400 MHz, $CDCl_3$) and ^{13}C NMR (125 MHz, $CDCl_3$) of tetraethylene glycol bromoisobutyrate initiator (TEGBriB).

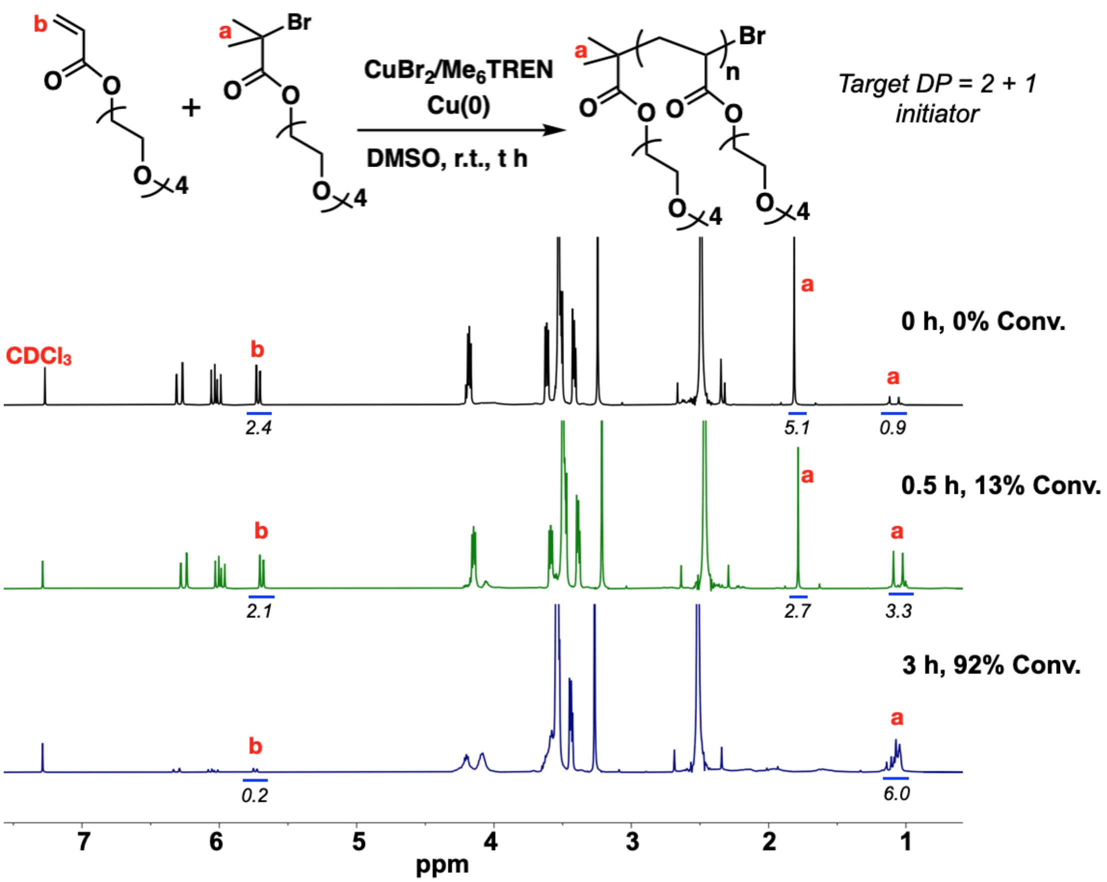

Figure S3. ^{1}H NMR (400 MHz, $CDCl_3$) of a few aliquots collected at intervals for ATRP of TEG-Ac with TEGBriB initiator

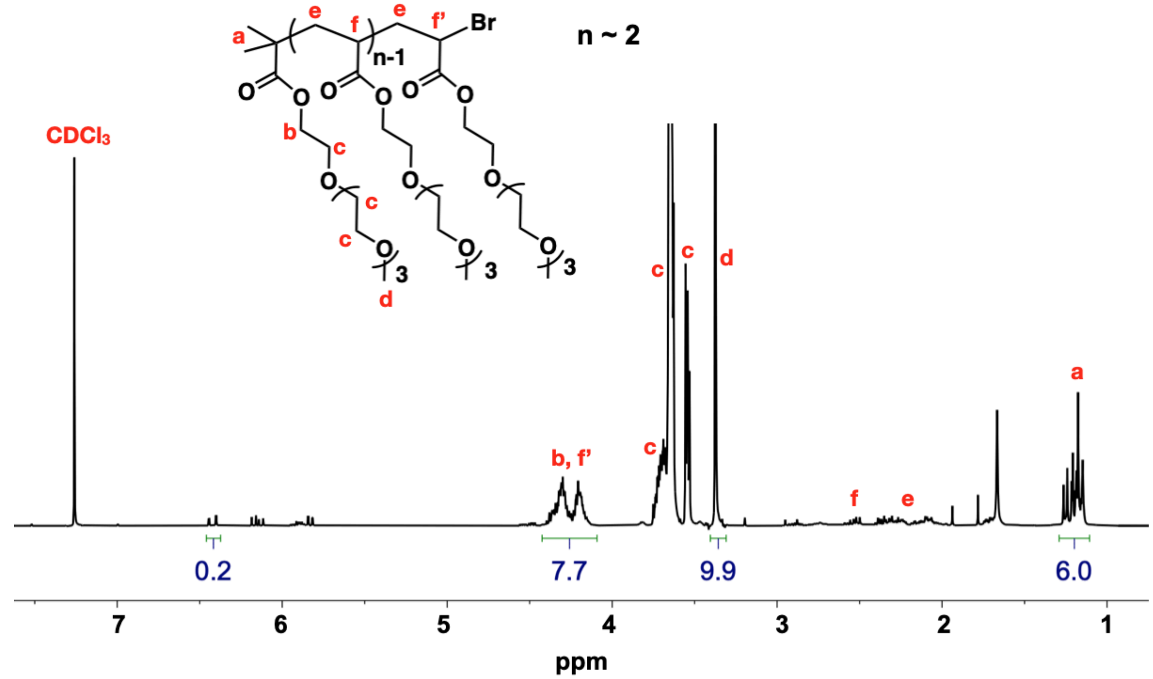

Figure S4. ^{1}H NMR (400 MHz, $CDCl_3$) of disperse ω-bromo oligo(tetraethylene glycol) $oTEG_3$-Br

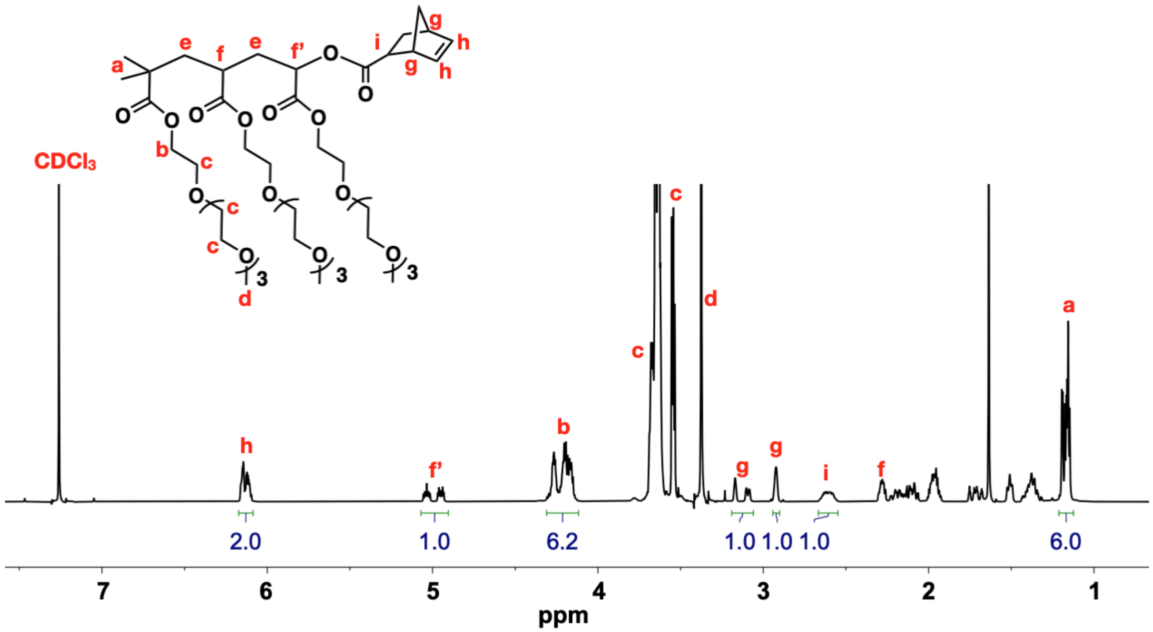

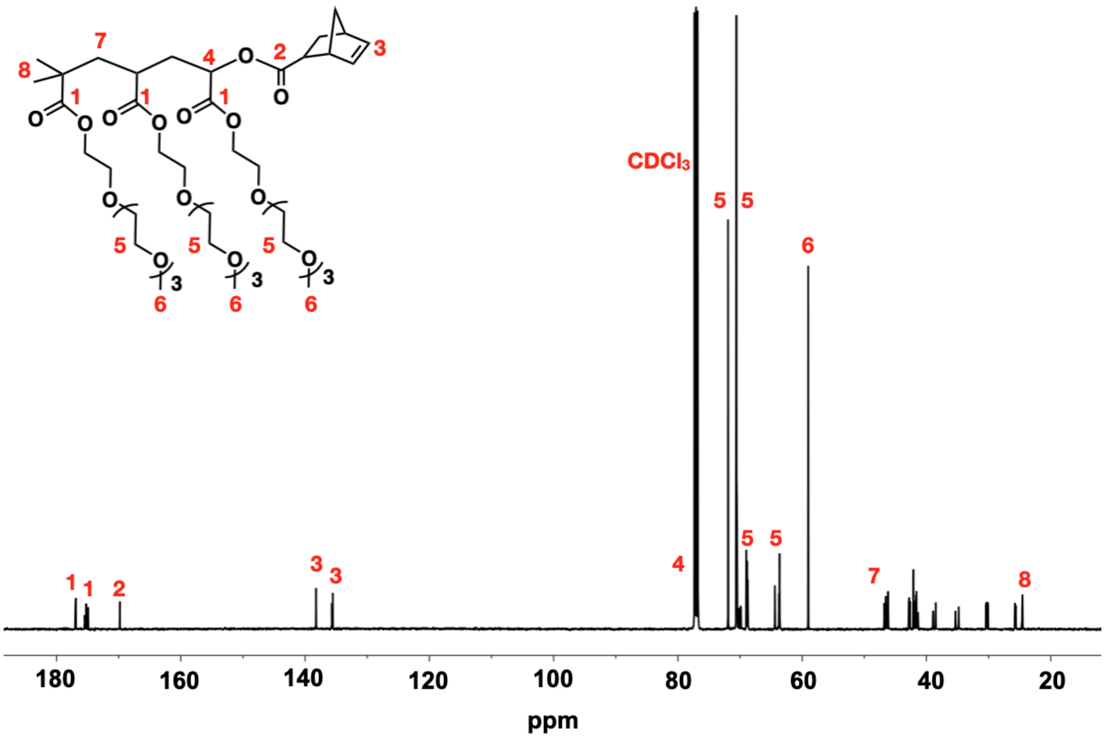

Figure S5. ^{1}H NMR (400 MHz, $CDCl_3$) and ^{13}C NMR (125 MHz, $CDCl_3$) of discrete ω-norbornenyl oligo(tetraethylene glycol) NB-oTEG$_3$ (**TE3**) isolated from disperse NB-oTEG$_3$

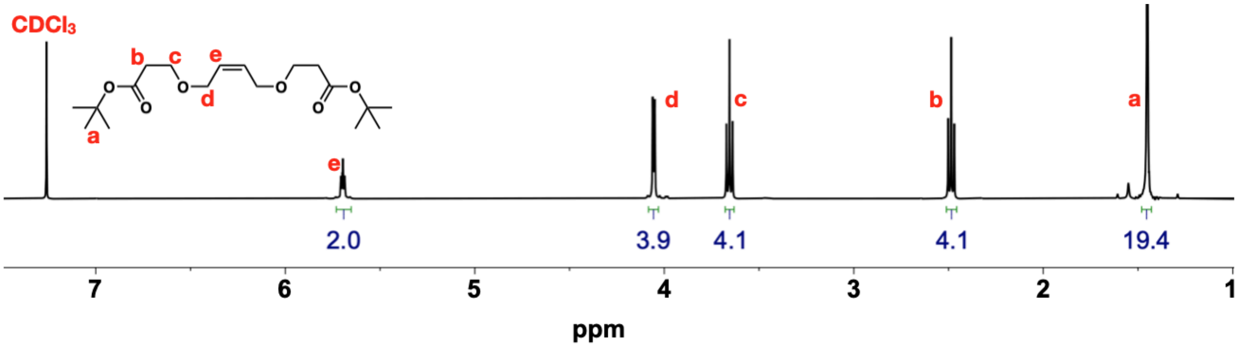

Figure S6. ^{1}H NMR (400 MHz, $CDCl_3$) of di-*tert*-butyl 3,3'-(but-2-ene-1,4-diylbis(oxy))(Z)-dipropionate

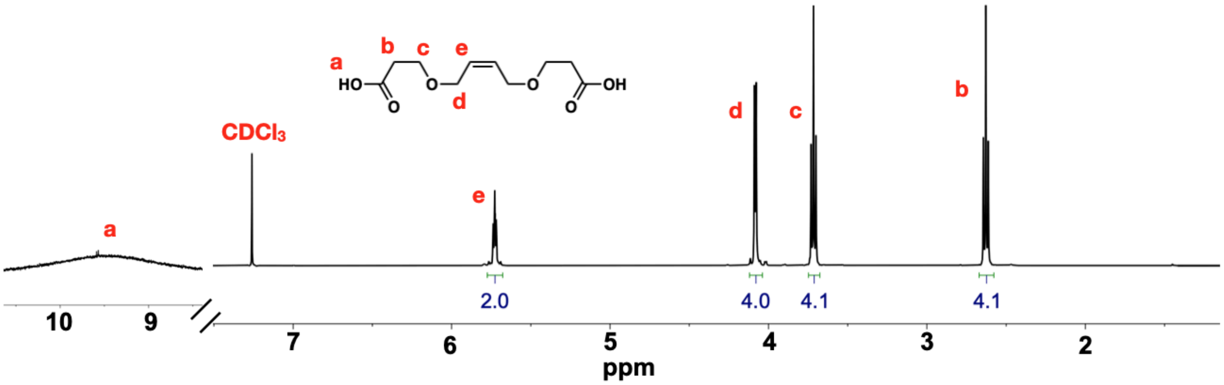

Figure S7. ^{1}H NMR (400 MHz, $CDCl_3$) of di-carboxylic acid 3,3'-(but-2-ene-1,4-diylbis(oxy))(Z)-dipropionate.

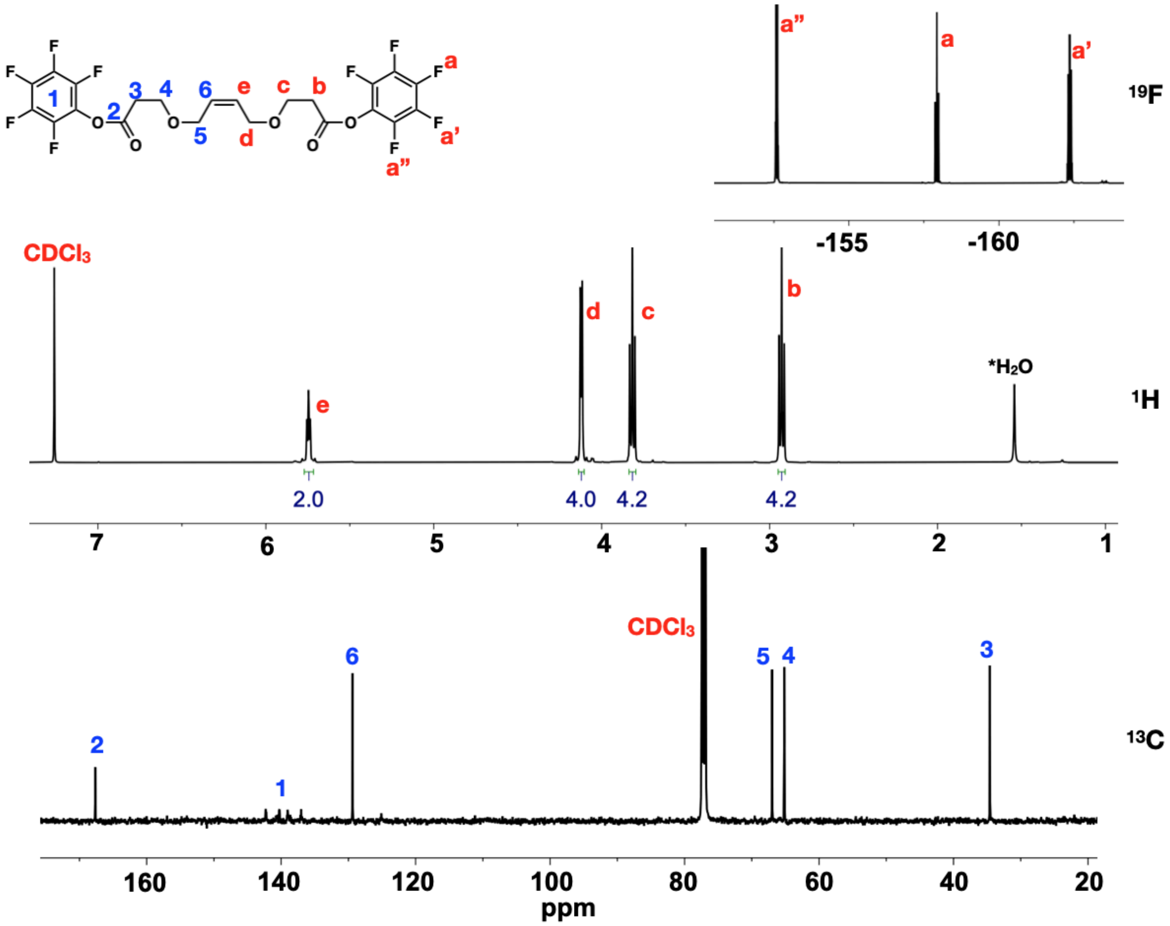

Figure S8. ^{19}F NMR (470 MHz), ^{1}H NMR (400 MHz), and ^{13}C NMR (125 MHz) of di-pentafluorophenyl 3,3'-(but-2-ene-1,4-diylbis(oxy))(Z)-dipropionate ROMP terminating agent (PFPTA) in $CDCl_3$**.**

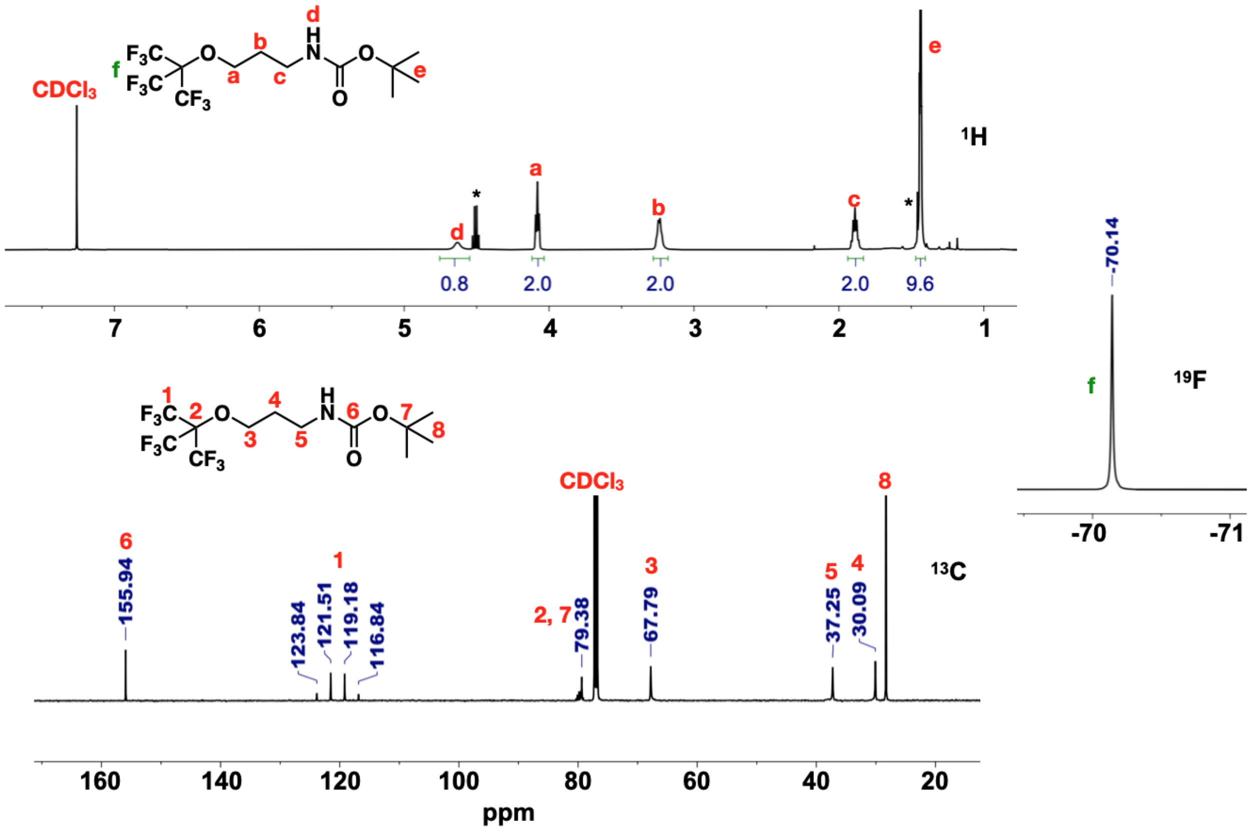

Figure S9. ^{19}F NMR (470 MHz), ^{1}H NMR (400 MHz), and ^{13}C NMR (125 MHz) of N-(tert-Butyloxycarbonyl)-3-amino-1-(nonafluoro-tert-butoxy)-propane in $CDCl_3$

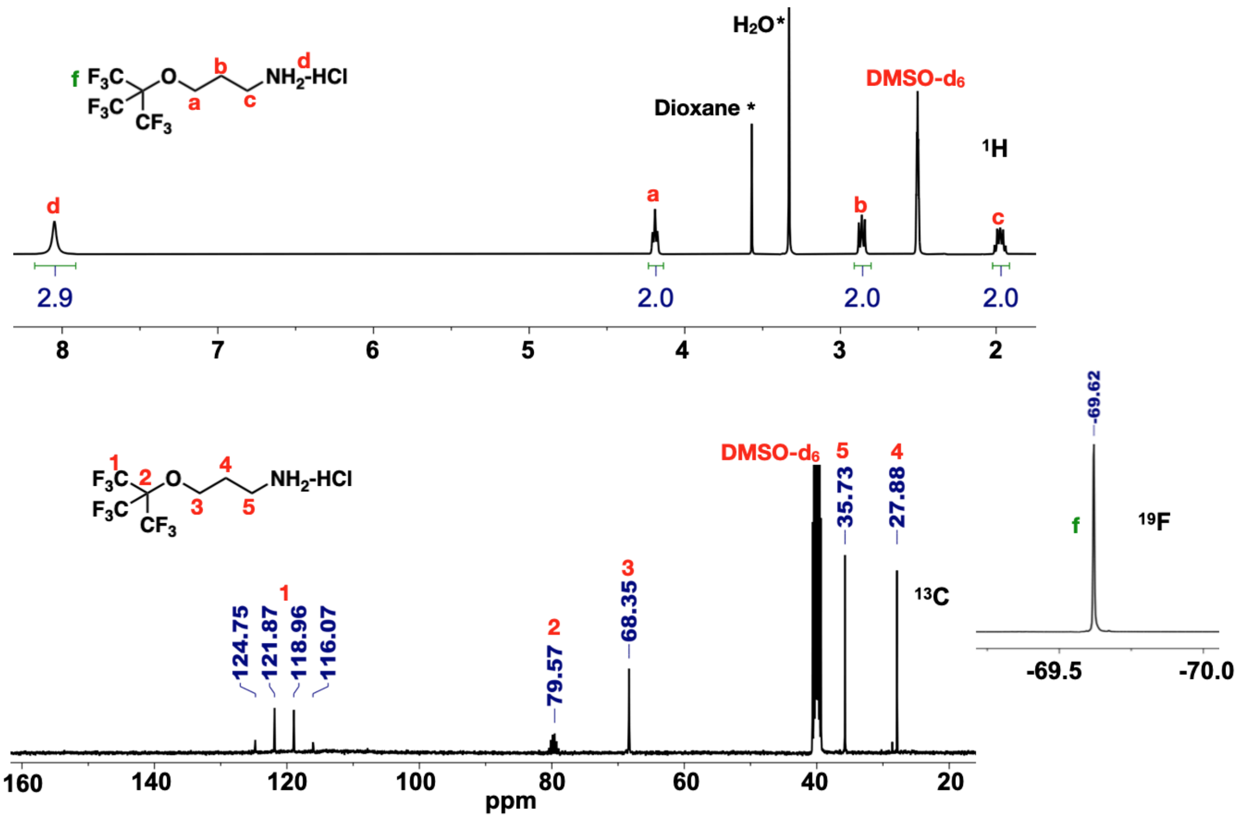

Figure S10. ^{19}F NMR (470 MHz), ^{1}H NMR (400 MHz), and ^{13}C NMR (125 MHz) of 3-amino-1-(nonafluoro-tert-butyoxy)propane hydrochloride in DMSO-d_6

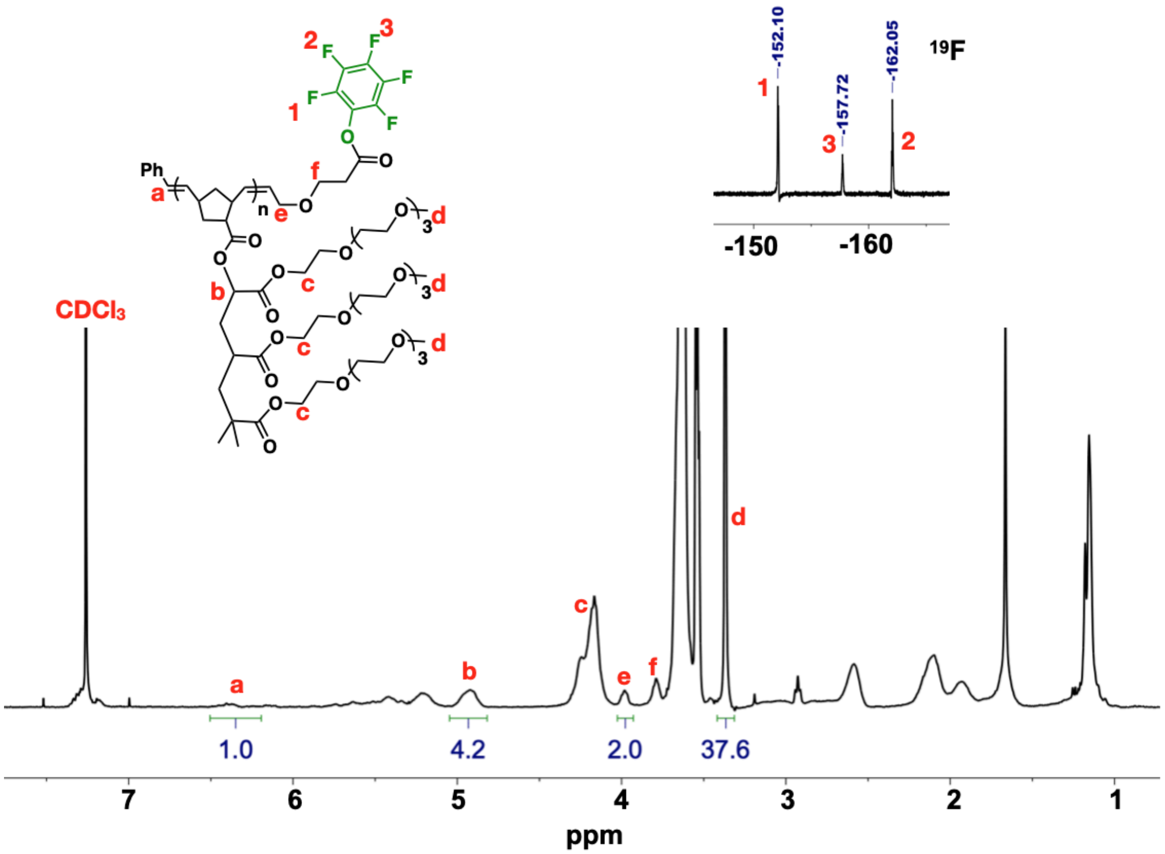

Figure S11. ^{19}F NMR (470 MHz) and ^{1}H NMR (500 MHz) of PFP-terminated precision bottlebrush polymer, **PBP-TE3$_5$-PFP** in $CDCl_3$.

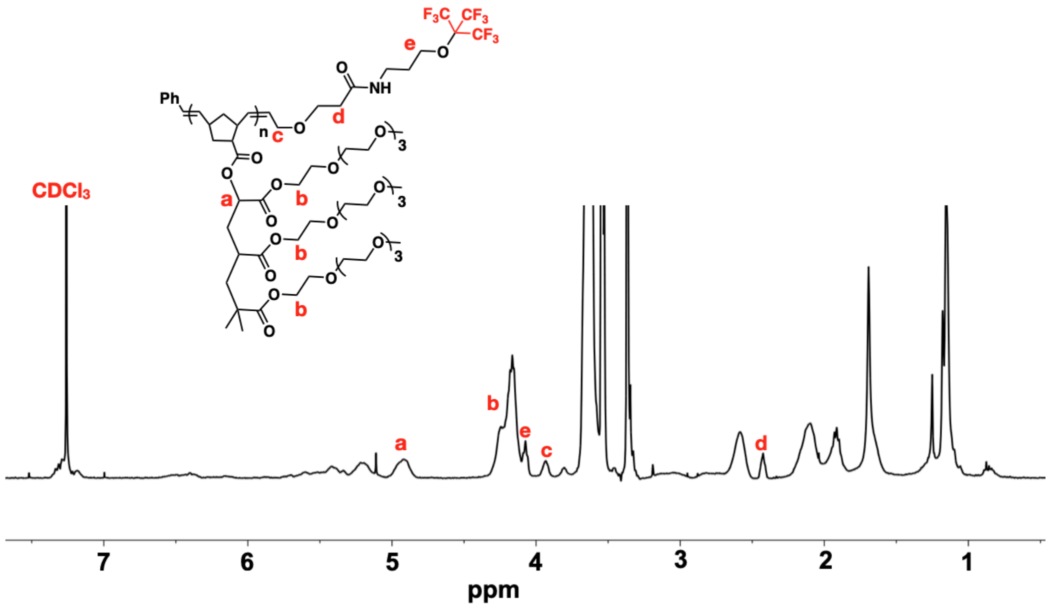

Figure S12. ^1H NMR (500 MHz, $CDCl_3$) of C_4F_9-terminated precision bottlebrush polymer, **PBF5**

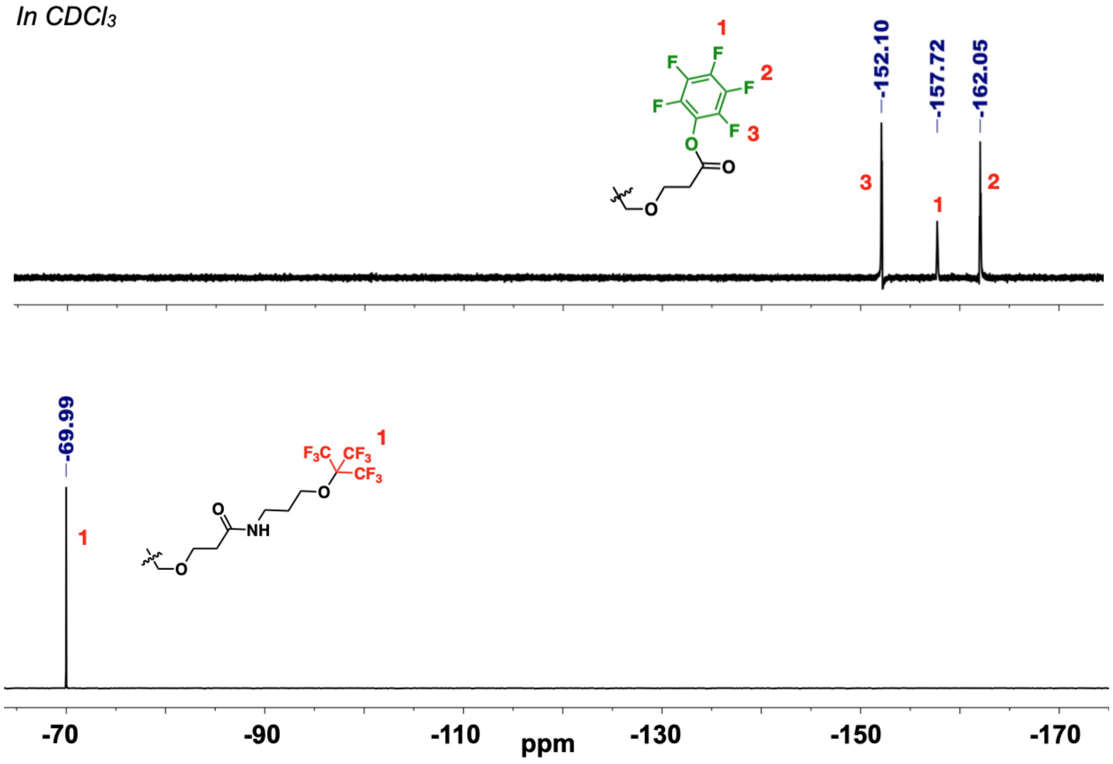

Figure S13. ^{19}F NMR (470 MHz, $CDCl_3$) spectra showing the conversion of **PBP-TE3$_5$-PFP** (top) to **PBF5** (bottom).

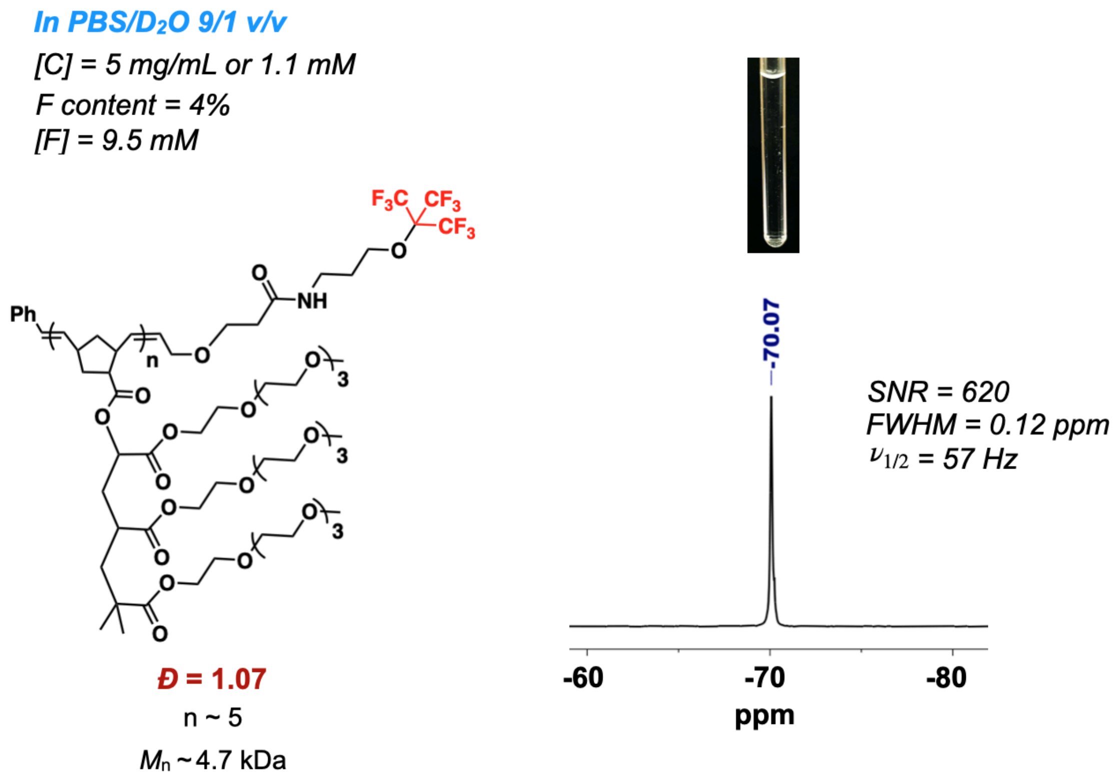

Figure S14. ^{19}F NMR (470 MHz) spectrum of 5 mg/mL **PBF5** in PBS/D_2O (9/1 v/v)

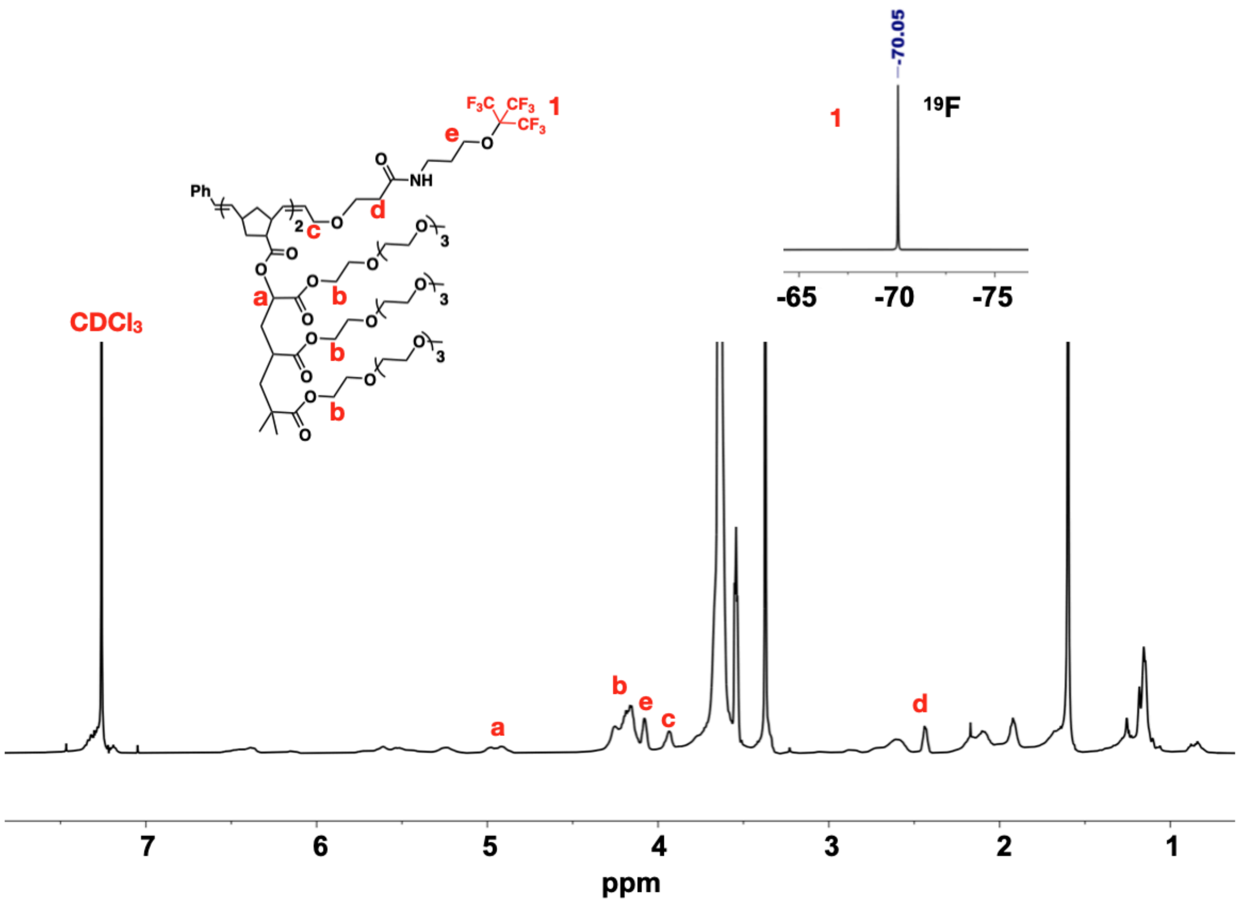

Figure S15. ^{19}F NMR (470 MHz) and ^{1}H NMR (500 MHz) of C_4F_9-terminated discrete bottlebrush polymer, **DBF2** in $CDCl_3$.

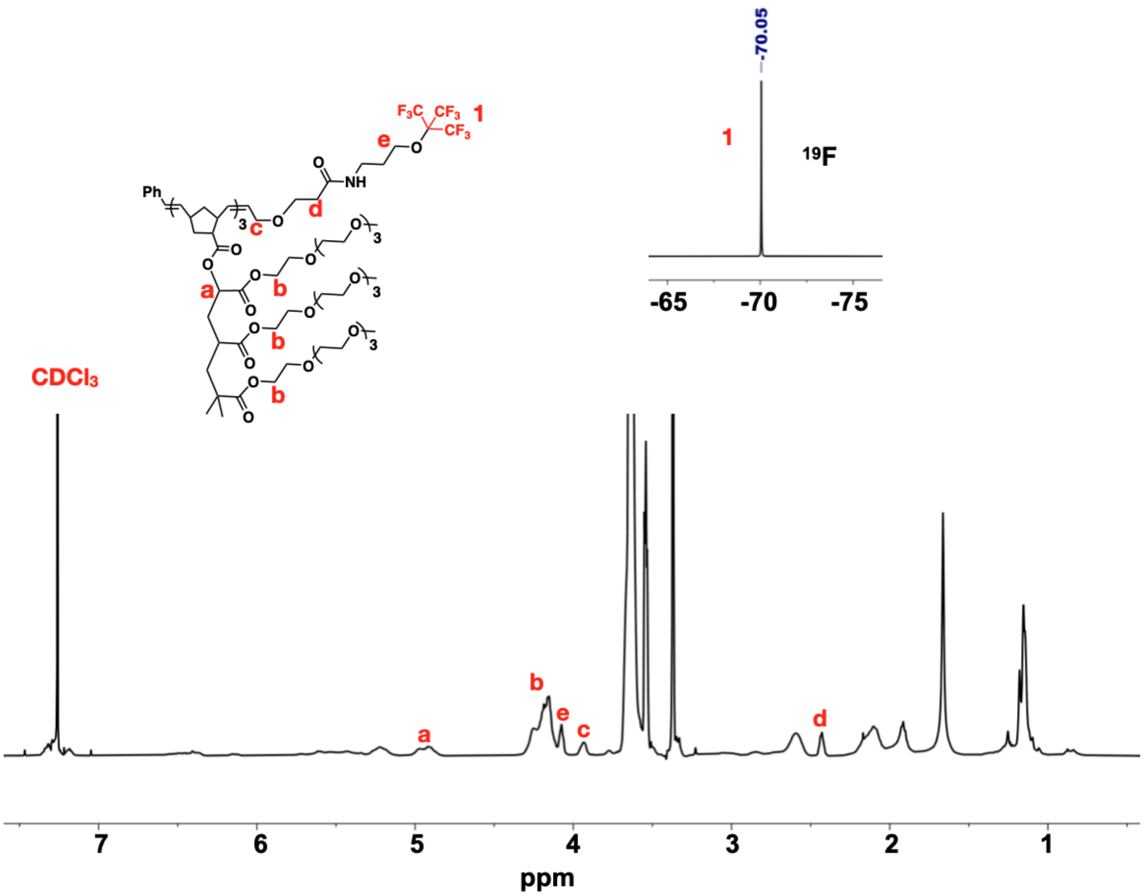

Figure S16. ^{19}F NMR (470 MHz) and ^{1}H NMR (500 MHz) of C_4F_9-terminated discrete bottlebrush polymer, **DBF3** in $CDCl_3$.

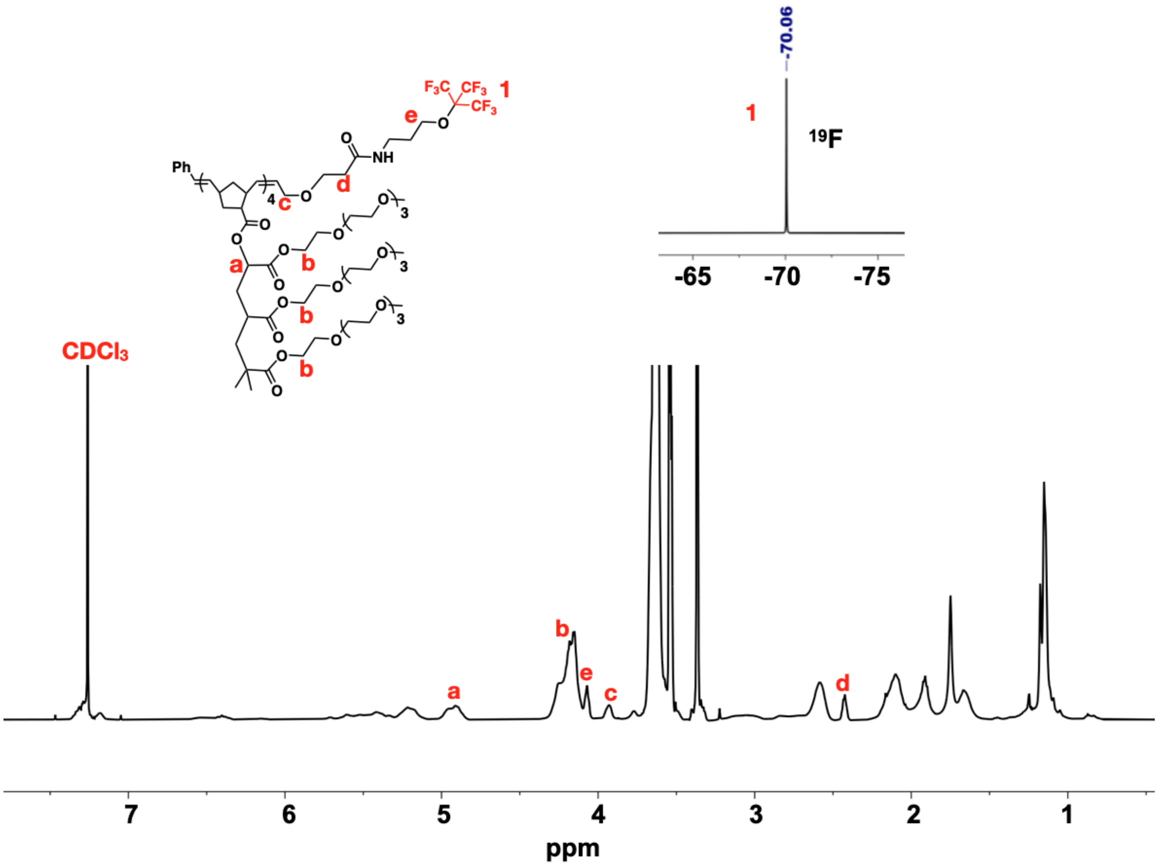

Figure S17. ^{19}F NMR (470 MHz) and ^{1}H NMR (500 MHz) of C_4F_9-terminated discrete bottlebrush polymer, **DBF4** in $CDCl_3$.

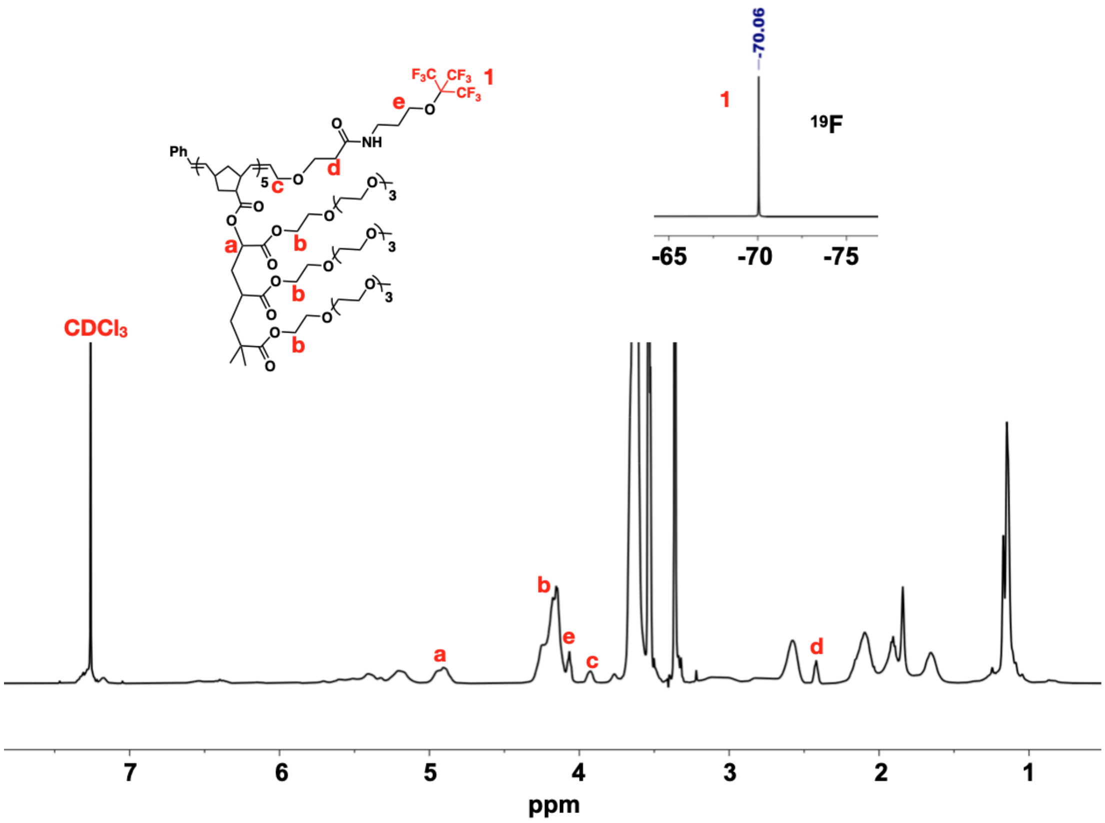

Figure S18. ^{19}F NMR (470 MHz) and ^{1}H NMR (500 MHz) of C_4F_9-terminated discrete bottlebrush polymer, **DBF5** in $CDCl_3$.

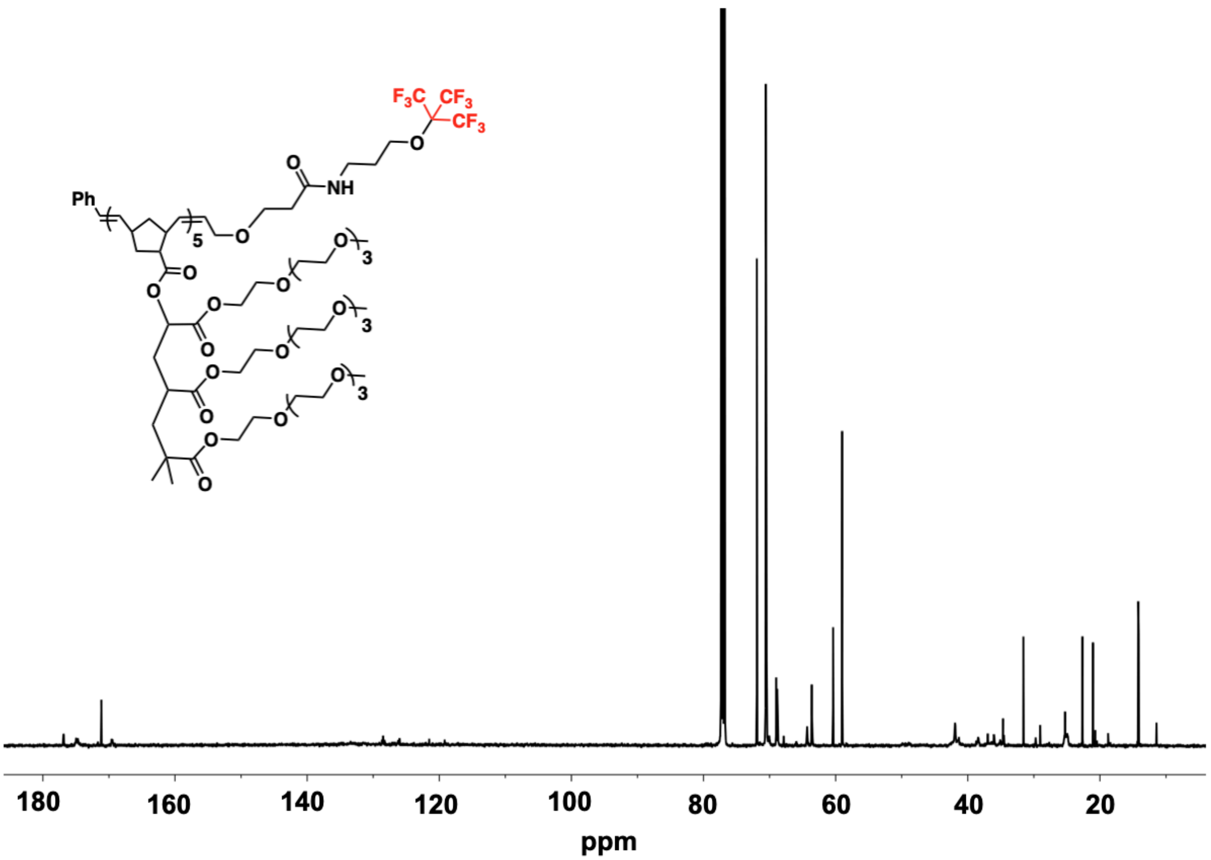

Figure S19. ^{13}C NMR (125 MHz) spectrum of C_4F_9-terminated discrete bottlebrush polymer, **DBF5** in $CDCl_3$.

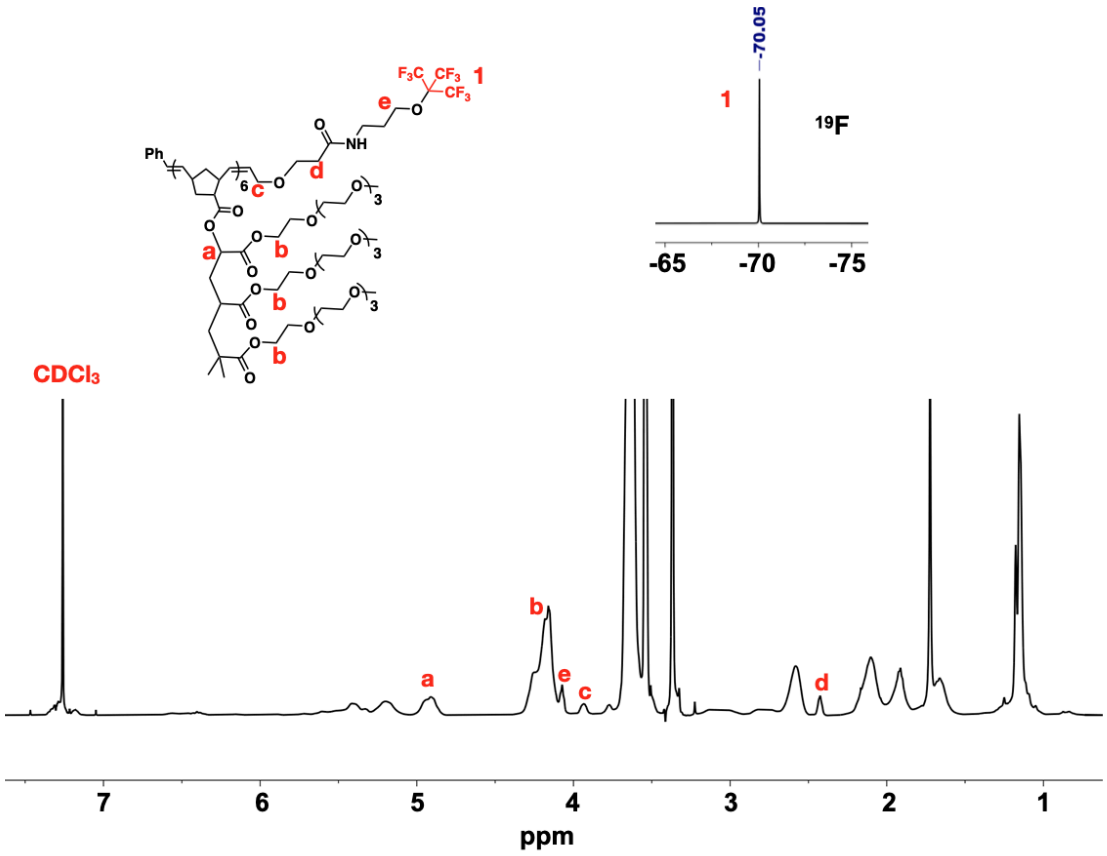

Figure S20. ^{19}F NMR (470 MHz) and ^{1}H NMR (500 MHz) of C_4F_9-terminated discrete bottlebrush polymer, **DBF6** in $CDCl_3$.

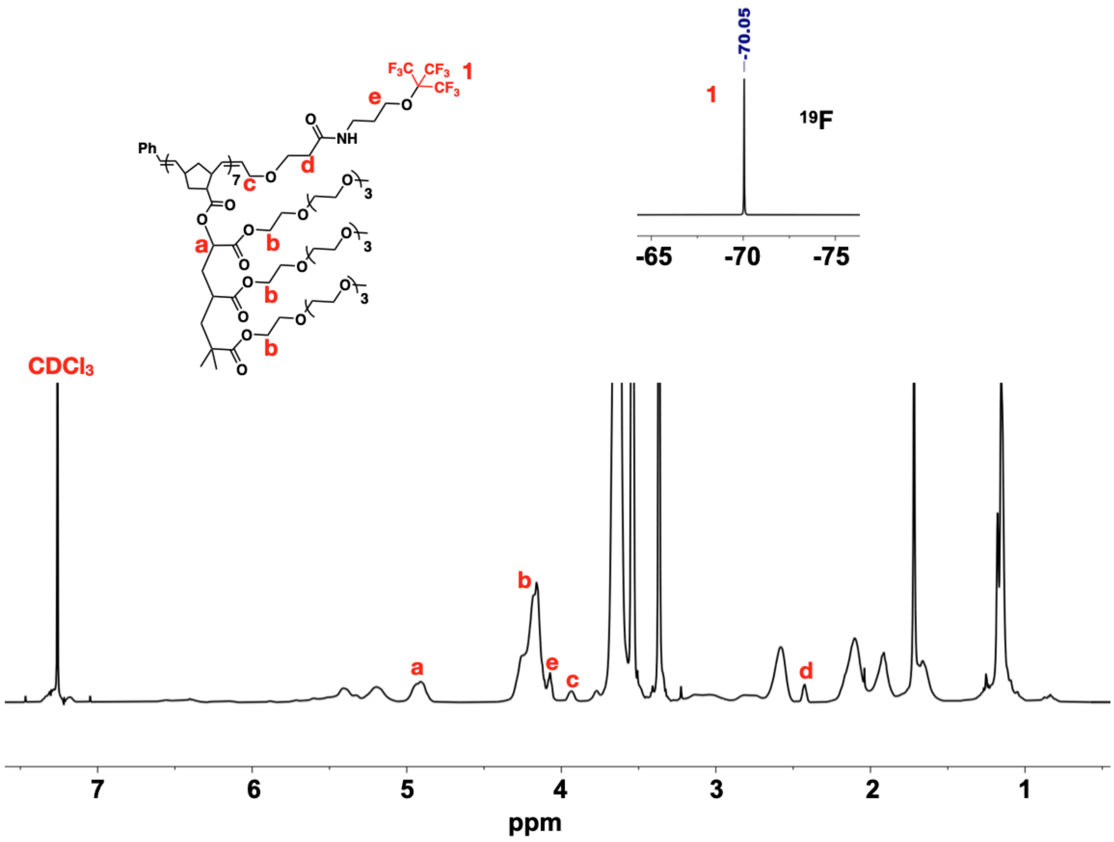

Figure S21. ^{19}F NMR (470 MHz) and ^{1}H NMR (500 MHz) of C_4F_9-terminated discrete bottlebrush polymer, **DBF7** in $CDCl_3$.

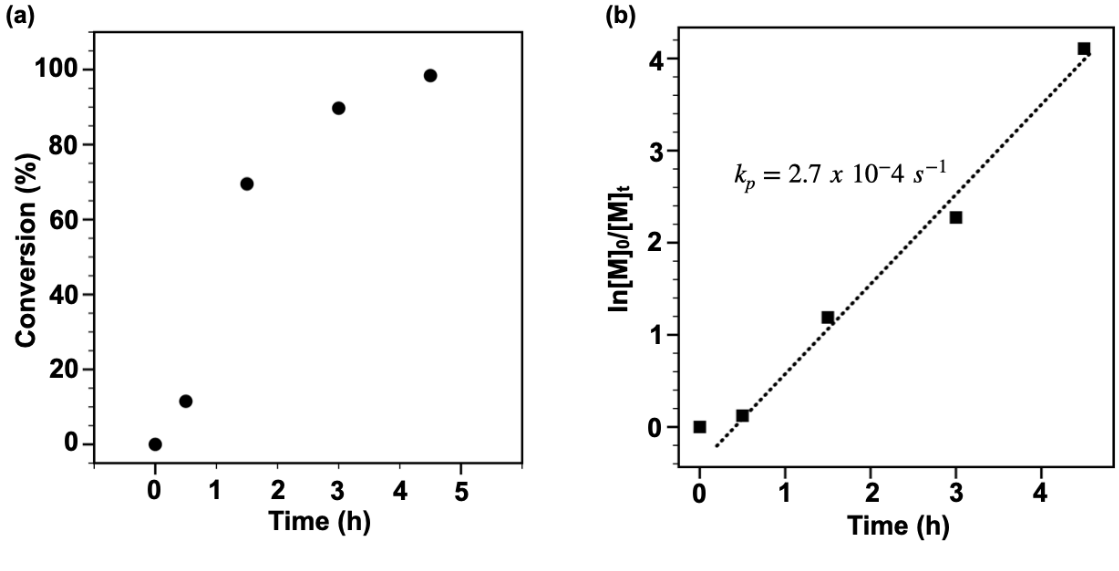

Figure S22. (a) Conversion of TEG-Ac monomer over time (b) In$[M]_0/[M]_t$ vs polymerization time for ATRP of TEG-Ac using TEGBriB.

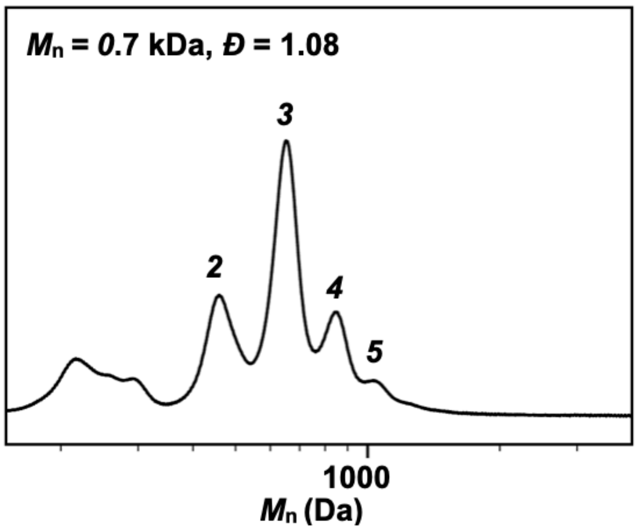

Figure S23. SEC profile of disperse **oTEG$_3$-Br**.

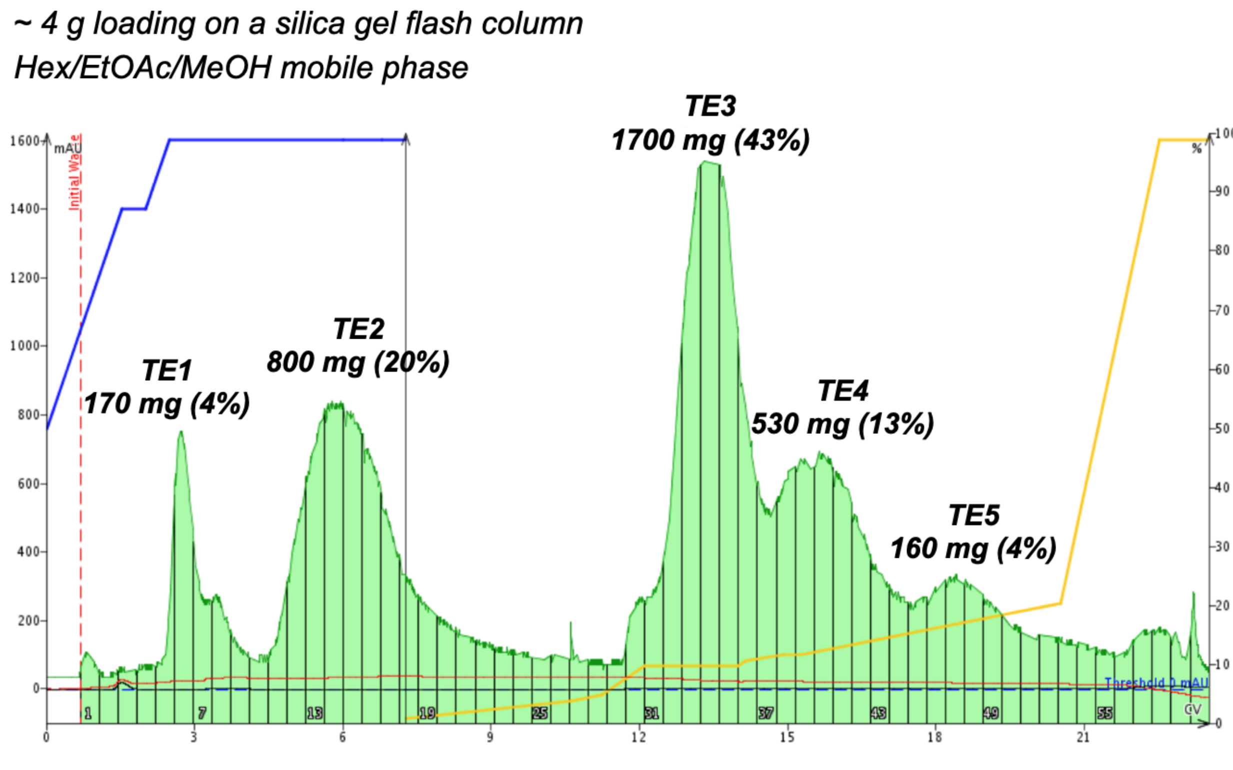

Figure S24. Gradient flash chromatography profile showing separation of disperse **NB-oTEG$_3$** into discrete macromonomer libraries. TE# represents discrete **NB-oTEG$_{\#}$** and the mass yield of each discrete fraction is included.

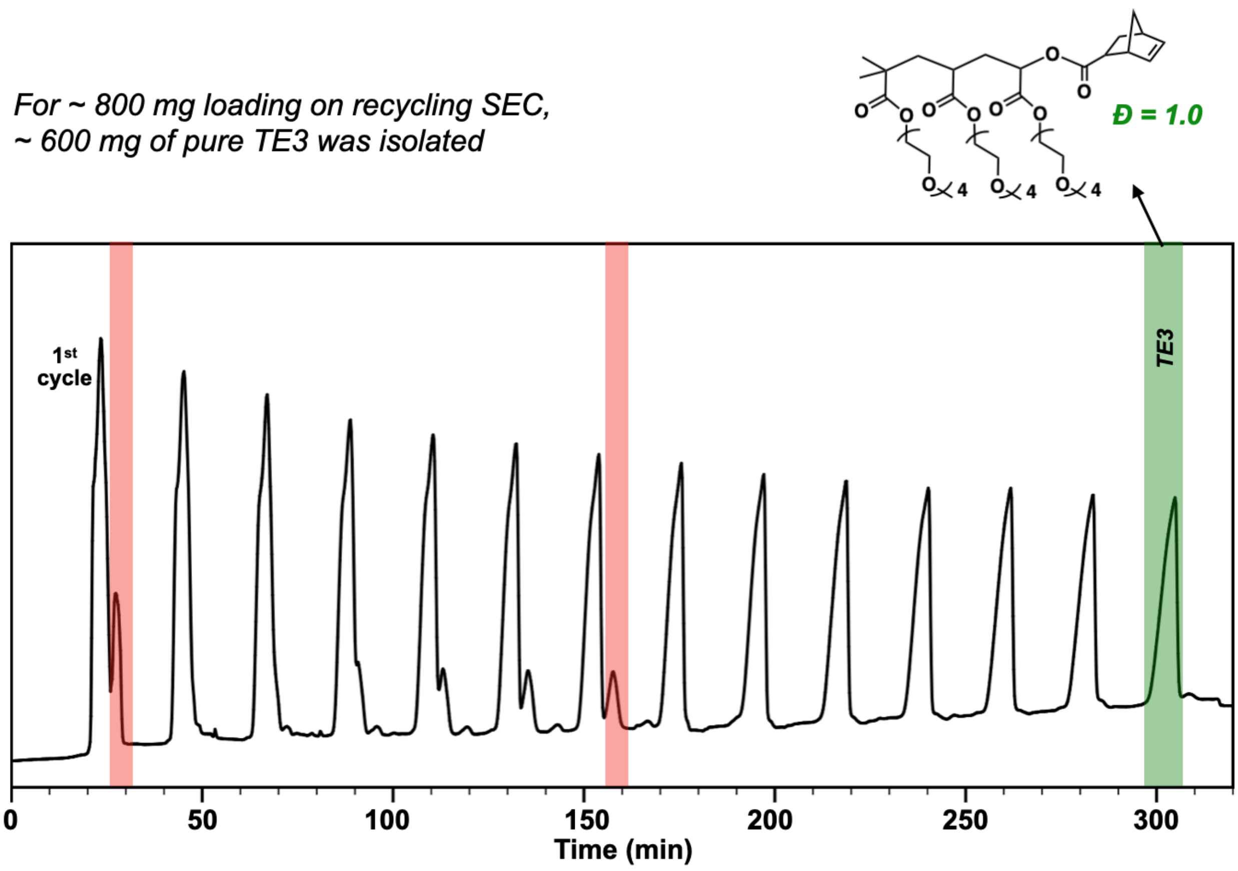

Figure S25. High resolution isolation of discrete **NB-oTEG$_3$** (TE3) using rSEC.

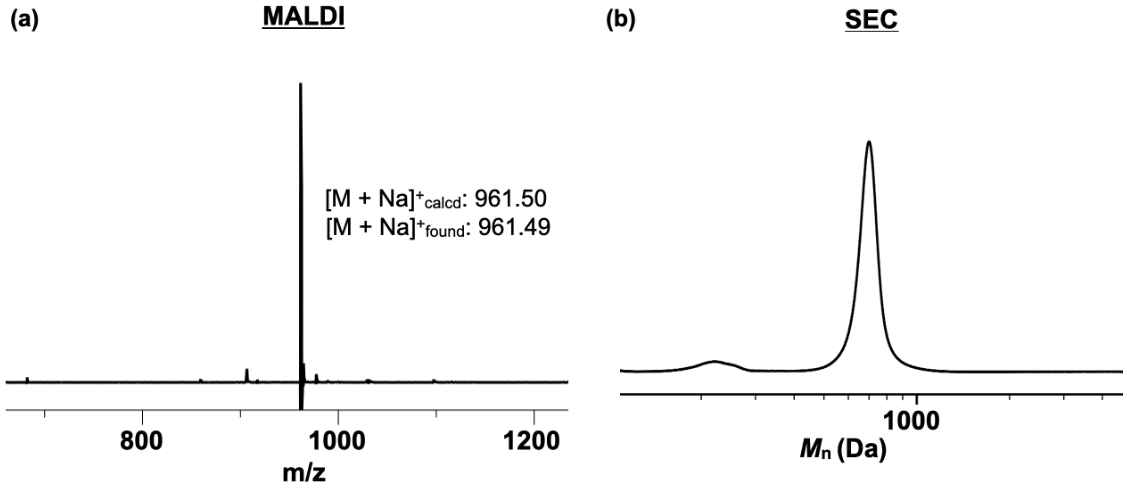

Figure S26. (a) MALDI-ToF spectrum (b) SEC profile of discrete **NB-oTEG$_3$** (TE3).

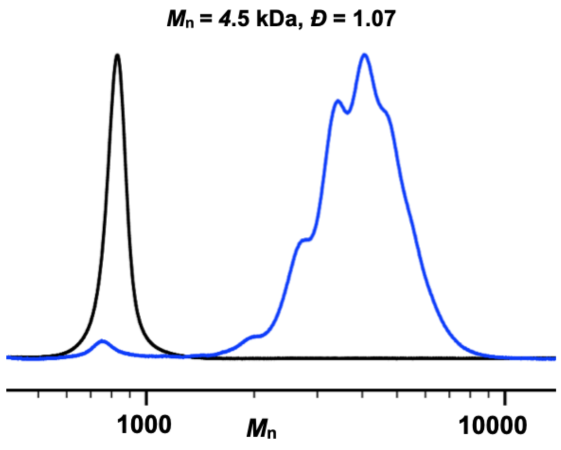

Figure S27. SEC profile of PFP-terminated precision bottlebrush polymer **PBP-TE3$_5$-PFP** (blue) from **TE3** macromonomer (black)

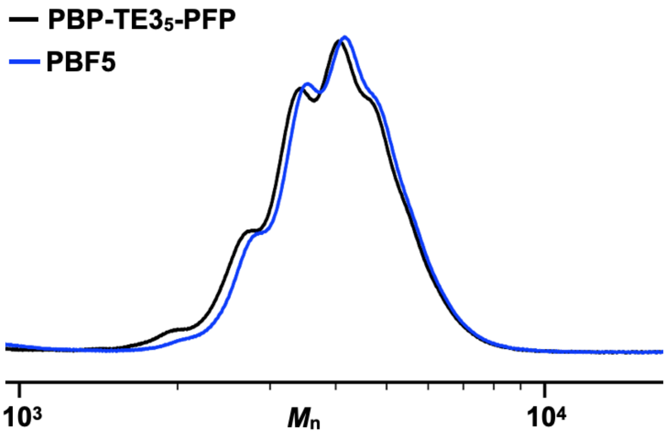

Figure S28. SEC profile showing the conversion of **PBP-TE3$_5$-PFP** (black) to **PBF5** (blue)

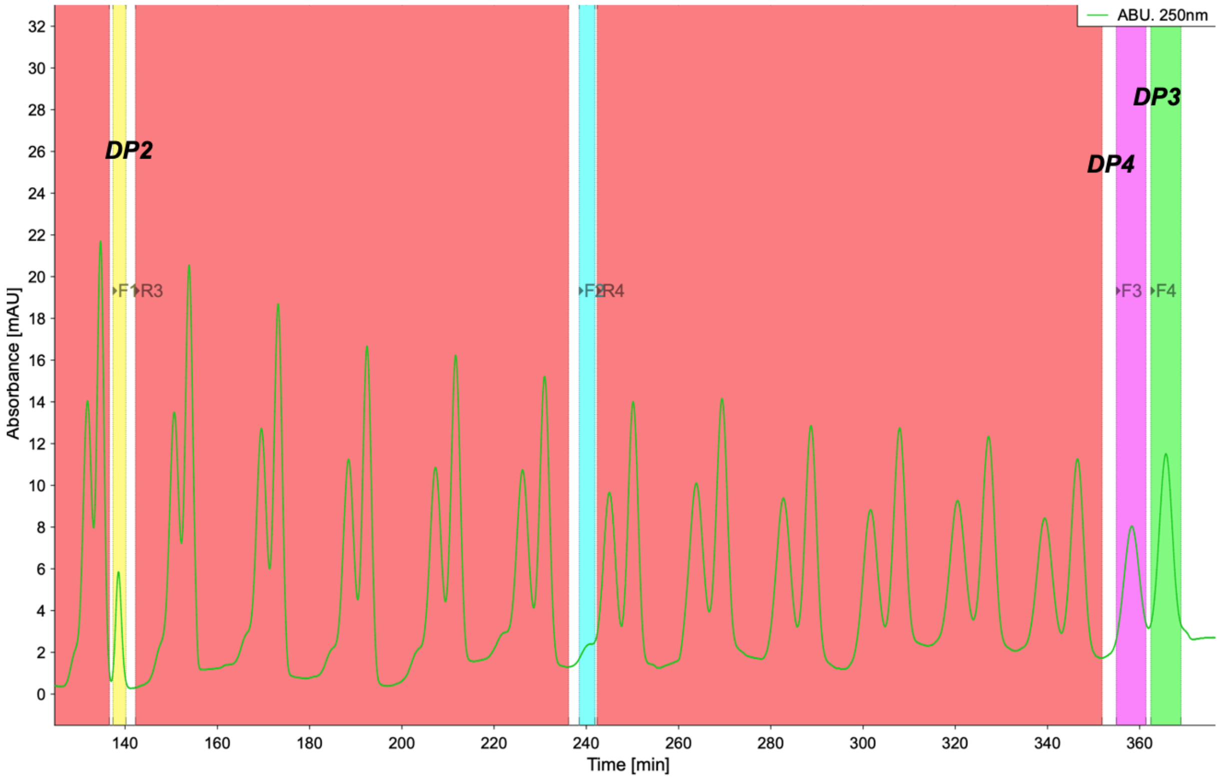

Figure S29. rSEC profile showing the isolation of **DBF2-4** from one batch of **PBF5** gotten from flash chromatography

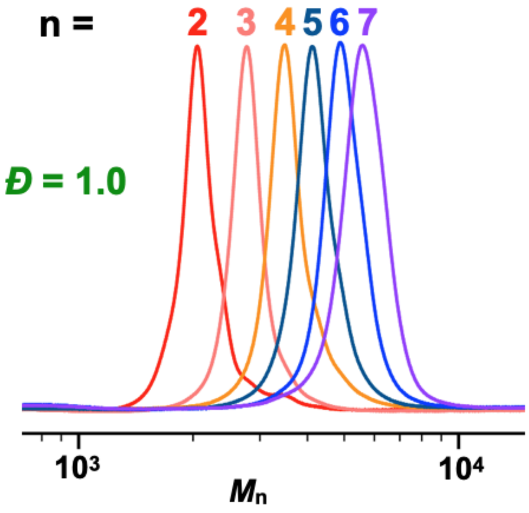

Figure S30. SEC profile of isolated discrete **DBFn** libraries

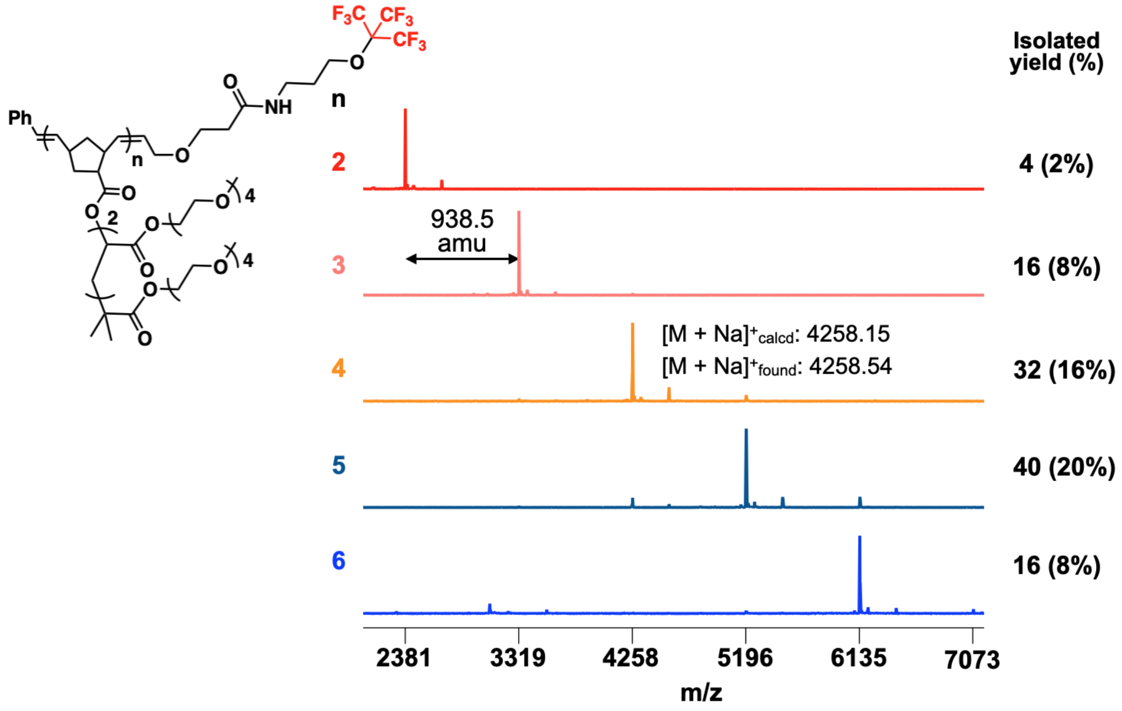

Figure S31. MALDI-ToF spectra of isolated discrete **DBFn** libraries

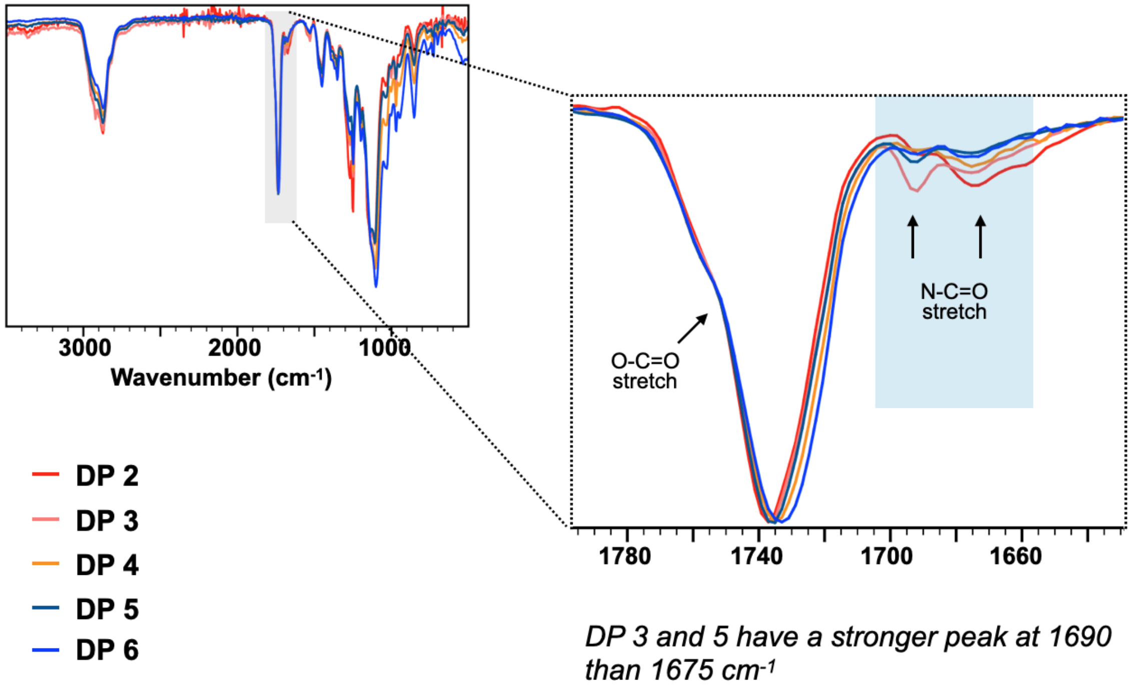

Figure S32. FTIR spectra of isolated discrete **DBFn** libraries

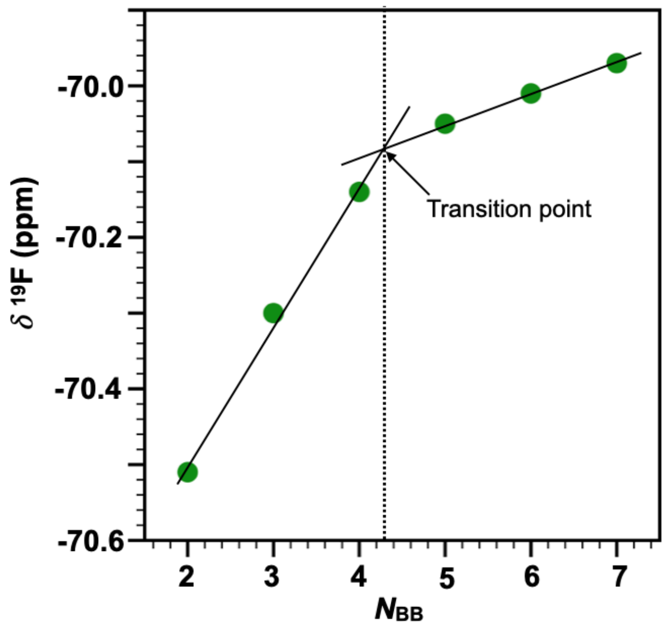

Figure S33. Plot of ^{19}F NMR chemical shift vs N_{BB} for discrete **DBFn** libraries.

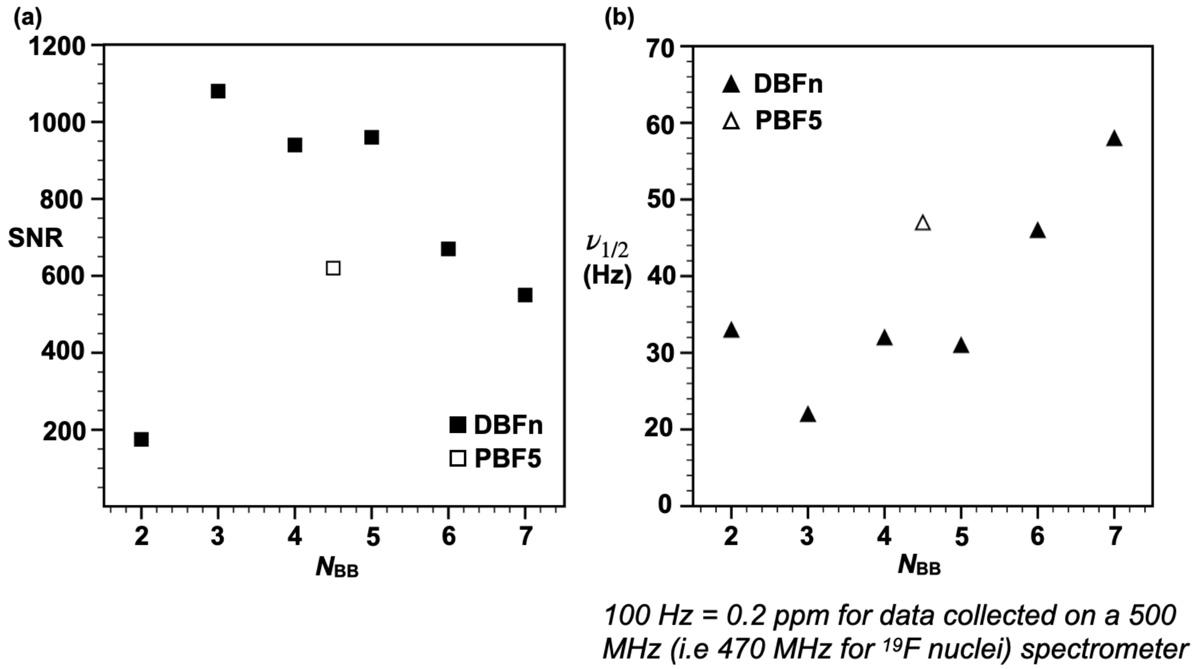

Figure S34. Plot of ^{19}F NMR (a) SNR and (b) chemical shift vs N_{BB} for discrete **DBFn** (filled shape) and **PBF5** (open shape) libraries.

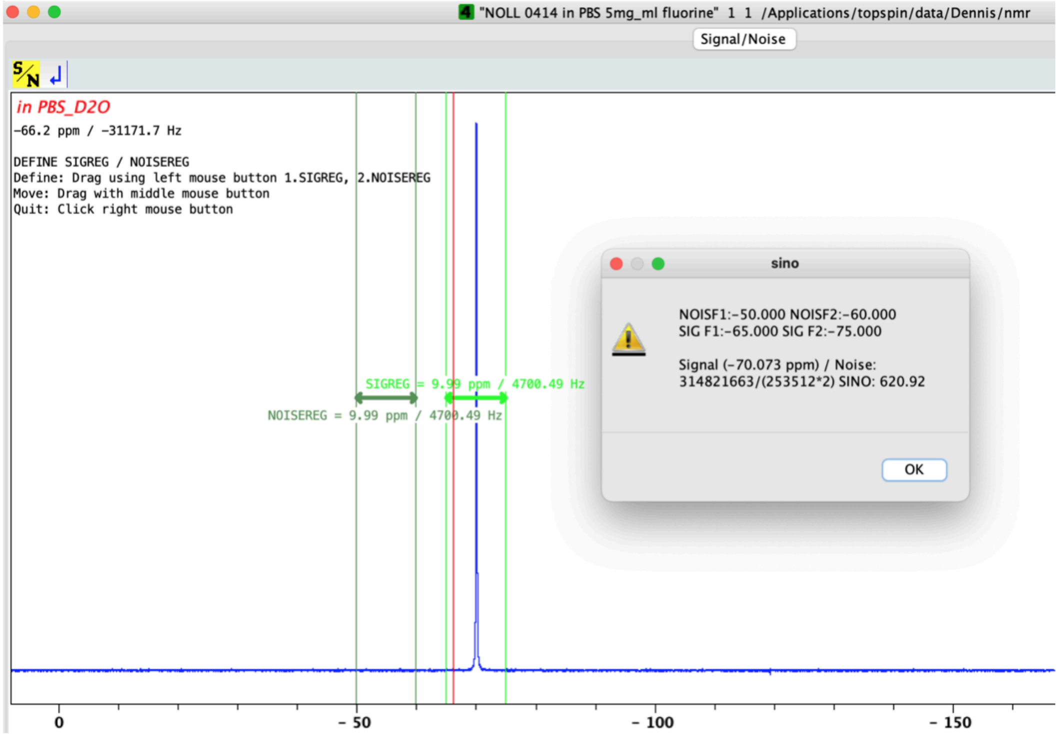

Figure S35. Example determination of ^{19}F NMR SNR of **PBF5** using Bruker Topspin software

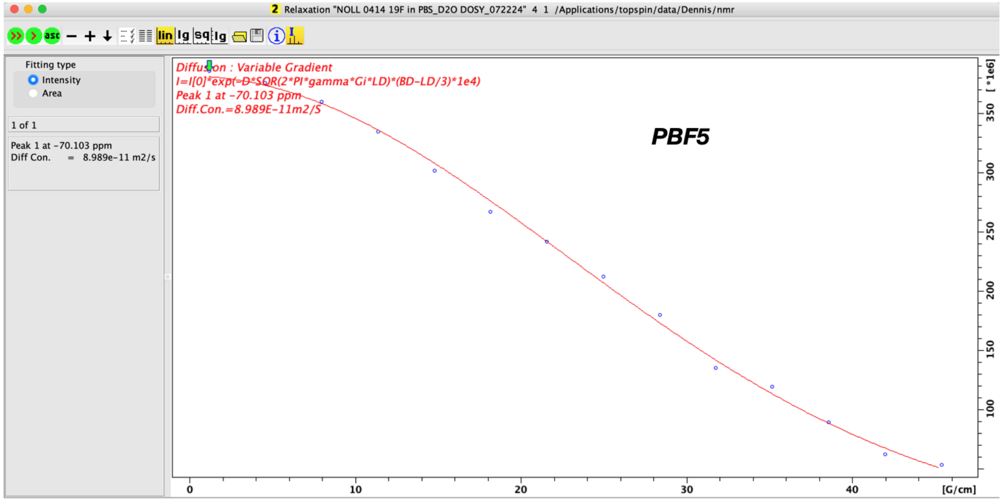

$$D_h = \frac{RT}{3N_A \pi \mu D} \qquad D_h = \frac{8.3 * 298}{3 * 6 * 10^{23} * \pi * 8.9 * 10^{-4} * 8.99 * 10^{-11}} = 5.5 * 10^{-9}\ m$$

for PBF5

Figure S36. Example ^{19}F DOSY NMR fitting of **PBF5** and determination of size (D_h)

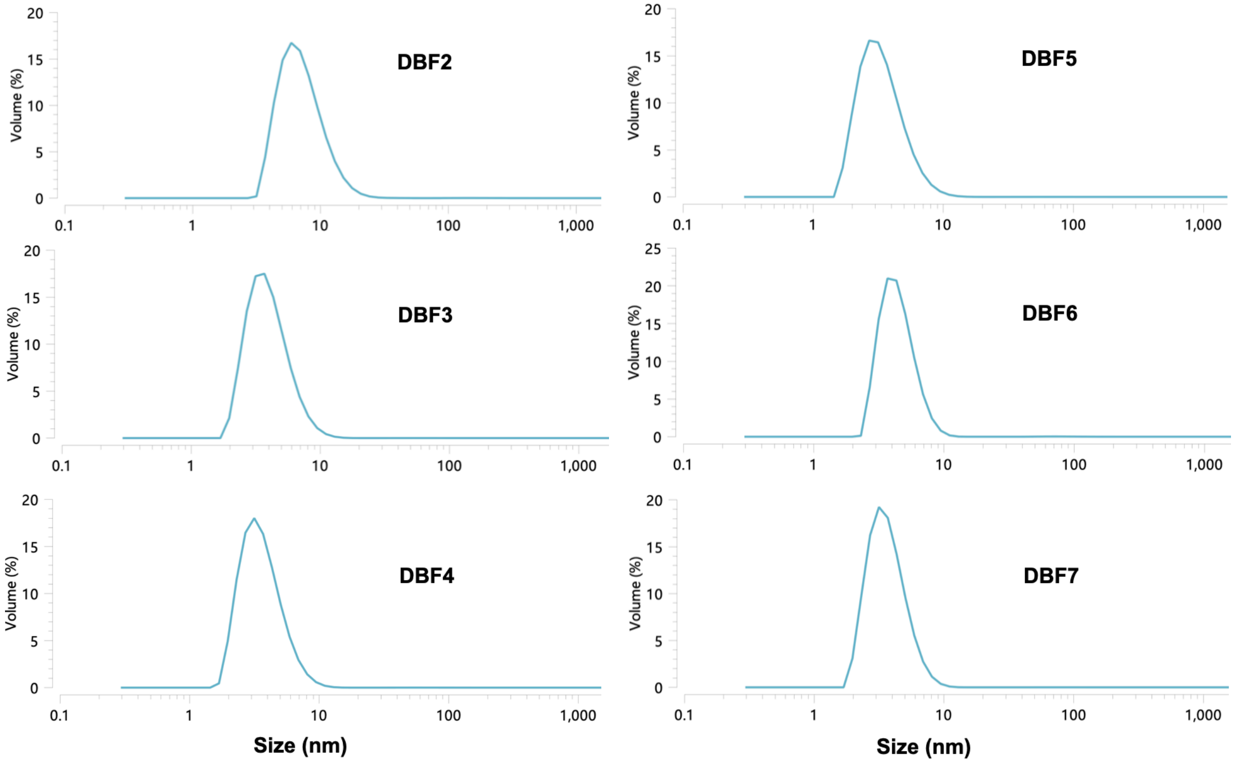

Figure S37. DLS volume distribution trace of discrete **DBFn** libraries in PBS/D_2O (5 mg/mL, 25 ºC)

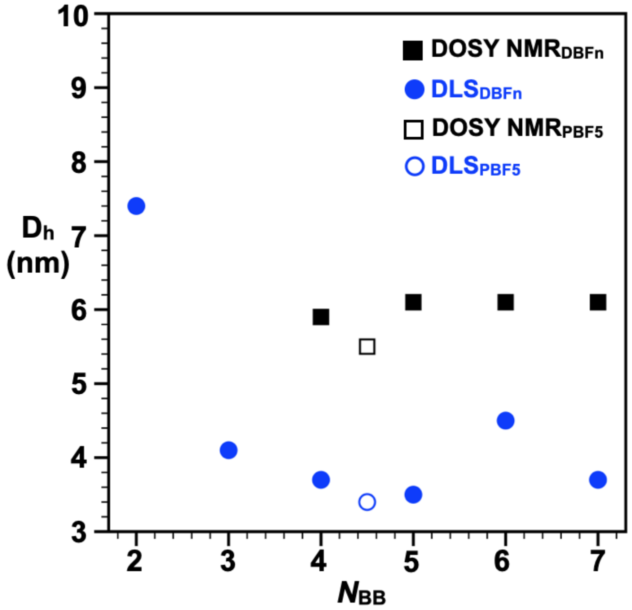

Figure S38. Plot of size vs N_{BB} of **DBFn** (filled shape) and **PBF5** (open shape) libraries. Data from ^{19}F DOSY NMR is in black, and DLS is in blue. Volume distribution data is reported for DLS

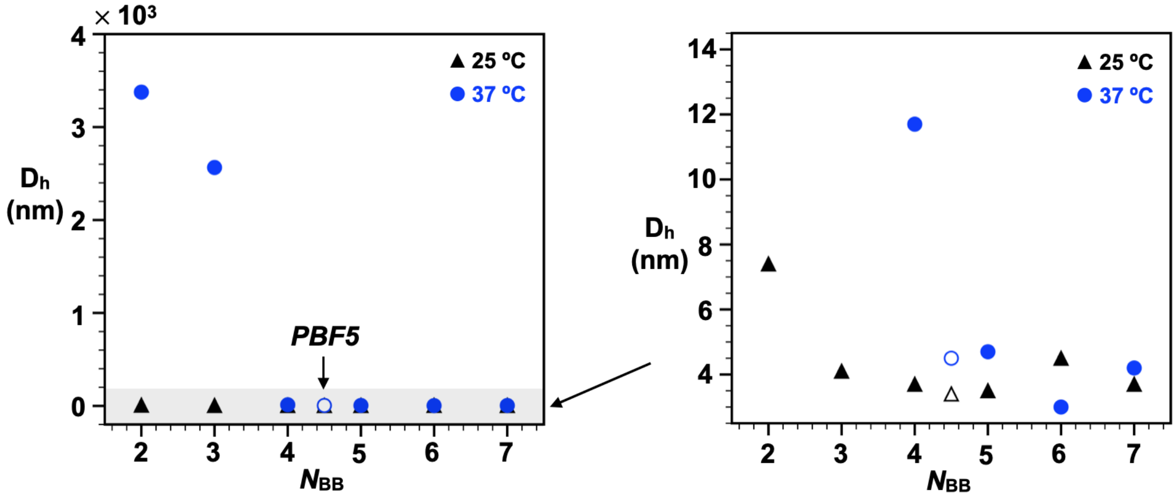

Figure S39. Plot of size vs N_{BB} of **DBFn** (filled shape) and **PBF5** (open shape) libraries at 25 ºC (black triangle) and 37 ºC (blue circle). Data from DLS volume distribution.

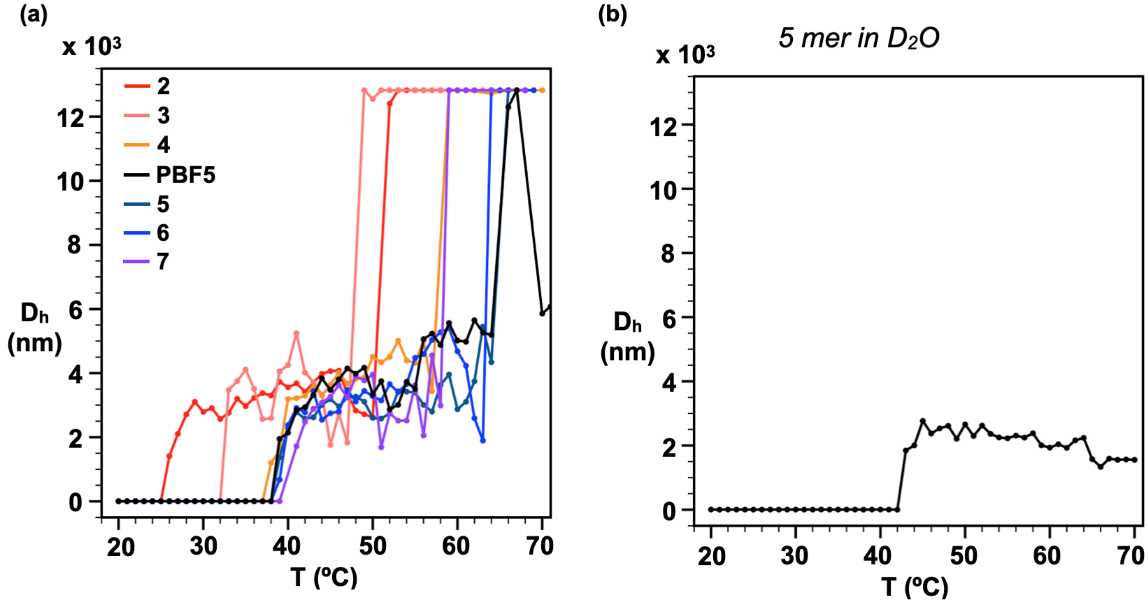

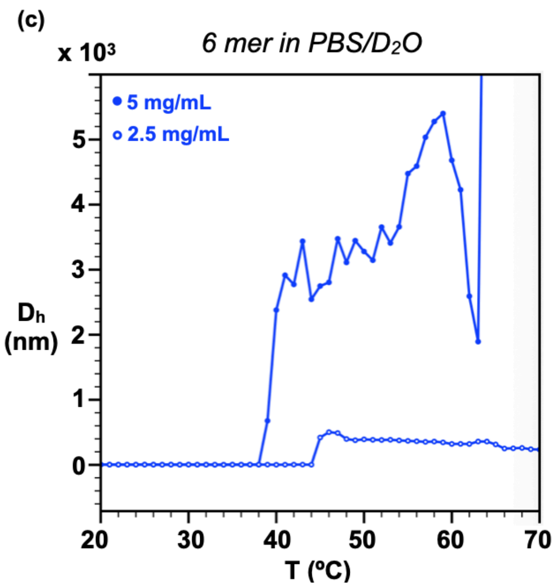

Figure S40. Size vs temperature trend of (a) DBFn and PBF5 libraries in PBS/D_2O (9/1 v/v) (b) DBF5 in D_2O, 2nd aggregation into >10 microns only observed in PBS/D_2O. (c) DBF6 in PBS/D_2O at 5 and 2.5 mg/mL.

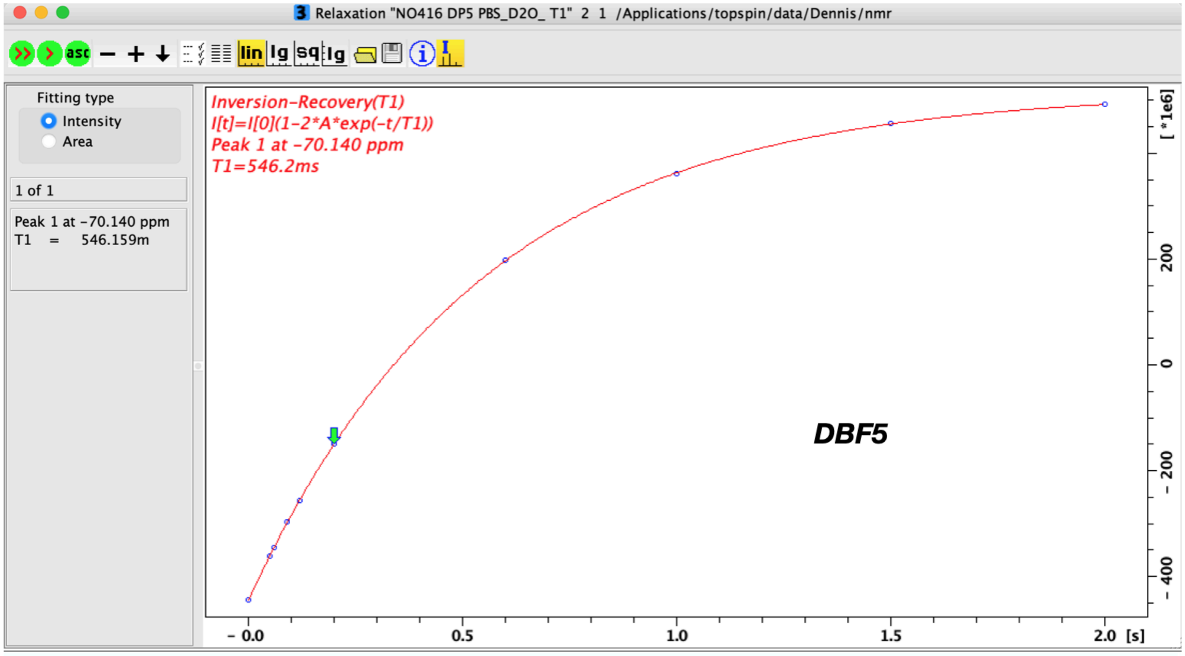

Figure S41. Example ^{19}F NMR T_1 relaxation fitting of **DBF5**

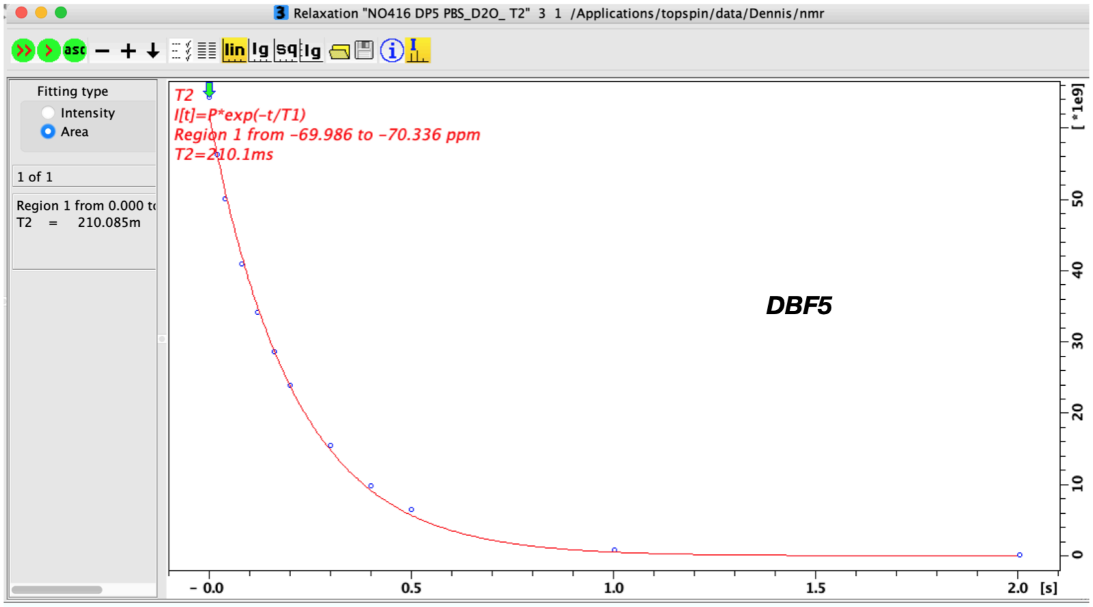

Figure S42. Example ^{19}F NMR T_2 relaxation fitting of **DBF5**

Table S1. ^{19}F NMR relaxation data of contrast agents

Sample[a]	$Đ$[b]	M_n[b] (kDa)	T_1 (ms)	T_2 (ms)	T_1/T_2
PBF5	1.07[c]	4.7[c]	560	211	2.7
DBF3	1.00	3.3	545	160	3.4
DBF5	1.00	5.2	546	210	2.6
DBF7	1.00	7.1	550	222	2.5

[a)] PB - precision bottlebrush with discrete sidechain and disperse backbone, DB - discrete bottlebrush polymer with discrete sidechain and backbone. The number represents the backbone length. All data were collected at a polymer concentration of 5 mg/mL in PBS/D_2O (9/1, v/v).
[b)] Determined from the MALDI-ToF experiment
[c)] Measured using SEC
T_1 & T_2 were determined from ^{19}F NMR relaxation experiments

16-Dec-24 | Compound Name: DBPS (1.2 mg) | Plate No:___1___

Assay: CTG Assay (A549)

Performed by/Date: ____16 Dec 2024____________ | Incubation Period : ___________48 hr_______

Microplate Orientation

									Control wells			
	1	2	3	4	5	6	7	8	9	10	11	12
A												
B		1.2 mg/ml	1.2 mg/ml	1.2 mg/ml	1.2 mg/ml	1.2 mg/ml	0.6 mg/ml	0.6 mg/ml	0.6 mg/ml	0.6 mg/ml	0.6 mg/ml	
C		0.3 mg/ml	0.3 mg/ml	0.3 mg/ml	0.3 mg/ml	0.3 mg/ml	0.15 mg/ml	0.15 mg/ml	0.15 mg/ml	0.15 mg/ml	0.15 mg/ml	
D		0.075 mg/ml	0.075 mg/ml	0.075 mg/ml	0.075 mg/ml	0.075 mg/ml	0.0375 mg/ml	0.0375 mg/ml	0.0375 mg/ml	0.0375 mg/ml	0.0375 mg/ml	
E		0.01875 mg/ml	0.01875 mg/ml	0.01875 mg/ml	0.01875 mg/ml	0.01875 mg/ml	0.009375 mg/ml	0.009375 mg/ml	0.009375 mg/ml	0.009375 mg/ml	0.009375 mg/ml	
F		SFM + A549	SFM + A549	SFM + A549	SFM + A549	SFM + A549	SFM + A549	SFM + A549	SFM + A549	SFM + A549	SFM + A549	
G		SFM + A549	SFM + A549	SFM + A549	SFM + A549	SFM + A549	SFM + A549	SFM + A549	SFM + A549	SFM + A549	SFM + A549	
H												

1

Cell line:____A549 (P#3)______________________________

Control wells	Serum Free Medium (SFM)+ A549

Time:________________

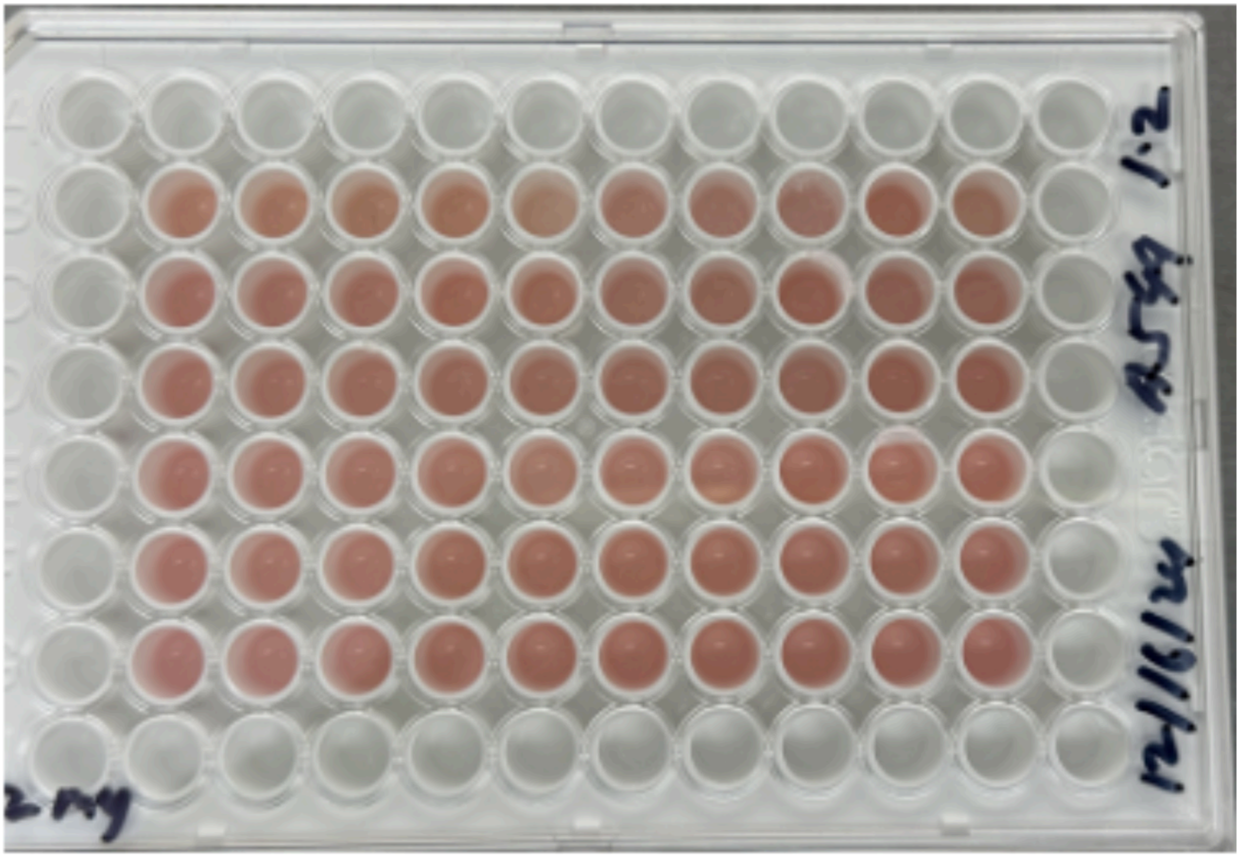

Figure S43. CTG assay on different concentrations of **DBF5**

Table S2. Cell count after exposure to **DBF5**

**Replicates**	**Vehicle Control wells**	**1.20 mg/ml**	**0.60 mg/ml**	**0.30 mg/ml**	**0.15 mg/ml**	**0.075 mg/ml**	**0.0375 mg/ml**	**0.01875 mg/ml**	**0.009375 mg/ml**
1	521574	529080	516736	560033	563905	581362	555163	566123	540616
2	571711	562045	572025	540972	563158	524854	552619	536332	560032
3	544490	566309	568177	556154	503801	548919	579613	546001	555824
4	551500	570453	567535	549885	569630	559796	520614	526872	534934
5	565945	546326	564292	546212	534997	542012	565778	565799	543506
6	551690								
7	563062								
8	556389								
9	532088								
10	565002								
**Average**	**552345.1**	**554842.6**	**557753**	**550651.2**	**547098.2**	**551388.6**	**554757.4**	**548225.4**	**546982.4**
**Percentage**	**100**	**100.452**	**100.979**	**99.693**	**99.050**	**99.827**	**100.437**	**99.254**	**99.029**

Table S3. Cell count after exposure to **PBF5**

**Replicates**	**Vehicle Control wells (SFM)**	**0.6 mg/ml**	**0.3 mg/ml**	**0.15 mg/ml**	**0.075 mg/ml**	**0.0375 mg/ml**	**0.01875 mg/ml**	**0.009375 mg/ml**	**0.0046875 mg/ml**	**Positive control (0.1% SDS)**
1	521574	281731	554769	548539	544437	562388	522816	548301	526771	1329
2	571711	259834	538154	522306	509709	579500	567084	577494	585579	1572
3	544490	237491	522252	526236	547831	522551	546255	551588	554305	1246
4	551500	314511	524080	576826	536171	543263	532769	516058	519324	1063
5	565945	346159	566488	512065	572847	513115	522868	550291	538540	
6	551690									
7	563062									
8	556389									
9	532088									
10	565002									
**Average**	**552345.1**	**287945.2**	**541148.6**	**537194.4**	**542199**	**544163.4**	**538358.4**	**548746.4**	**544903.8**	**1302.5**
**Percentage**	**100**	**52.131**	**97.973**	**97.257**	**98.163**	**98.519**	**97.468**	**99.348**	**98.653**	**0.452**

16-Dec-24

Compound Name: PBPS (0.6 mg)

Plate No:__1__

Assay: CTG Assay (A549)

Performed by/Date: 16 Dec 2024

Incubation Period : 48 hr

Microplate Orientation

	1	2	3	4	5	6	7	8	9	10	11	12
									Control wells			
A												
B		0.6 mg/ml	0.6 mg/ml	0.6 mg/ml	0.6 mg/ml	0.6 mg/ml	0.3 mg/ml	0.3 mg/ml	0.3 mg/ml	0.3 mg/ml	0.3 mg/ml	
C		0.15 mg/ml	0.15 mg/ml	0.15 mg/ml	0.15 mg/ml	0.15 mg/ml	0.075 mg/ml	0.075 mg/ml	0.075 mg/ml	0.075 mg/ml	0.075 mg/ml	
D		0.0375 mg/ml	0.0375 mg/ml	0.0375 mg/ml	0.0375 mg/ml	0.0375 mg/ml	0.01875 mg/ml	0.01875 mg/ml	0.01875 mg/ml	0.01875 mg/ml	0.01875 mg/ml	
E		0.009375 mg/ml	0.009375 mg/ml	0.009375 mg/ml	0.009375 mg/ml	0.009375 mg/ml	0.0046875 mg/ml	0.0046875 mg/ml	0.0046875 mg/ml	0.0046875 mg/ml	0.0046875 mg/ml	
F										0.1% SDS	0.1% SDS	
G										0.1% SDS	0.1% SDS	
H												

Positive control	0.1% SDS (20 µL of 10 % SDS and 1980 µL of SFM)

Cell line: A549 (P#3)

Time:

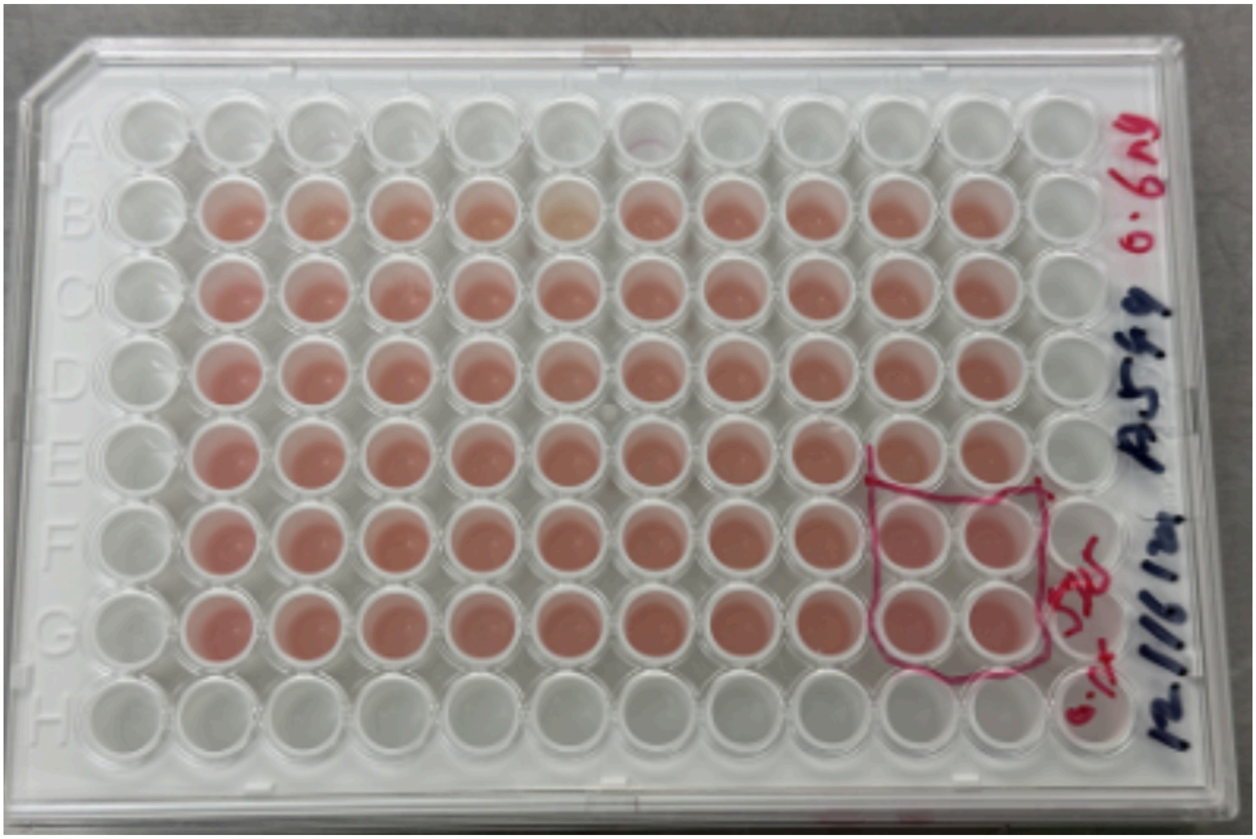

Figure S44. CTG assay on different concentrations of **PBF5.**

13-Mar-24

Compound Name: DBF3

Assay: CTG Assay (A549)

Performed by/Date: ______13 Mar 2024______________

Plate No:___1___

Incubation Period : ___________48 hr_______

Microplate Orientation

Control wells

	1	2	3	4	5	6	7	8	9	10	11	12
A												
B		0.6 mg/ml	0.6 mg/ml	0.6 mg/ml	0.6 mg/ml	0.6 mg/ml	0.3 mg/ml	0.3 mg/ml	0.3 mg/ml	0.3 mg/ml	0.3 mg/ml	
C		0.15 mg/ml	0.15 mg/ml	0.15 mg/ml	0.15 mg/ml	0.15 mg/ml	0.075 mg/ml	0.075 mg/ml	0.075 mg/ml	0.075 mg/ml	0.075 mg/ml	
D		0.0375 mg/ml	0.0375 mg/ml	0.0375 mg/ml	0.0375 mg/ml	0.0375 mg/ml	0.01875 mg/ml	0.01875 mg/ml	0.01875 mg/ml	0.01875 mg/ml	0.01875 mg/ml	
E		0.009375 mg/ml	0.009375 mg/ml	0.009375 mg/ml	0.009375 mg/ml	0.009375 mg/ml	0.0046875 mg/ml	0.0046875 mg/ml	0.0046875 mg/ml	0.0046875 mg/ml	0.0046875 mg/ml	
F		SFM	SFM	SFM	SFM	SFM	SFM	SFM	SFM	SFM	SFM	
G		SFM	SFM	SFM	SFM	SFM	SFM	SFM	SFM	SFM	SFM	
H												

Cell line:____A549 (P#3)______________________________

Control wells	Serum Free Medium (SFM) Only + No cells

Time:_______________

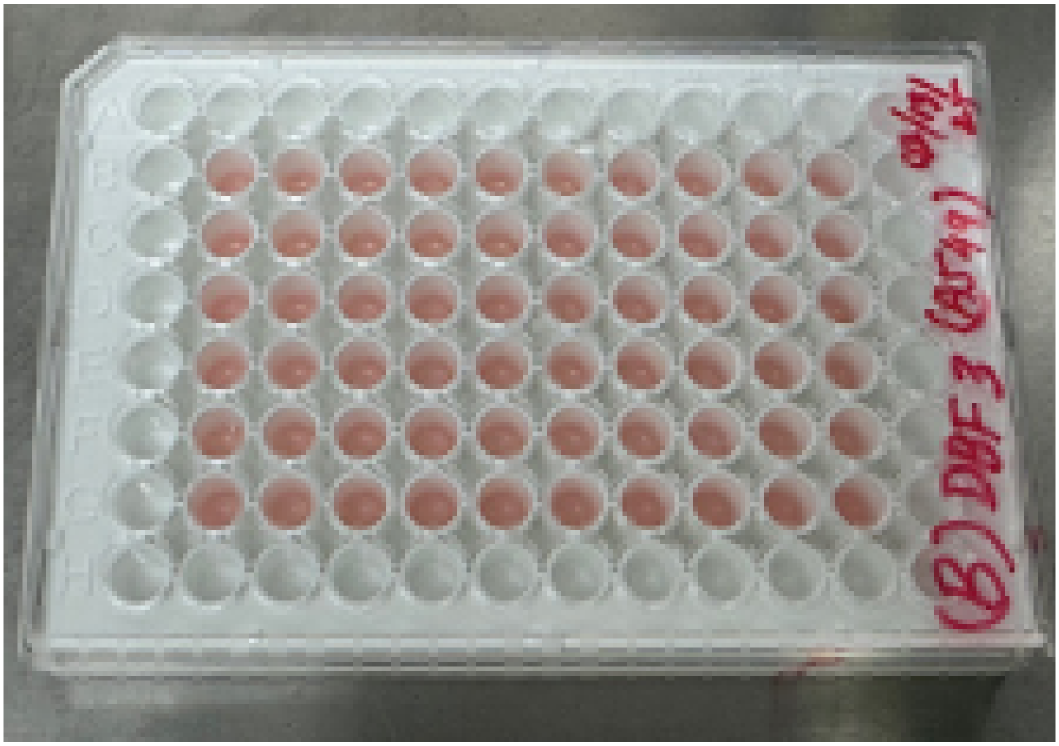

Figure S45. CTG assay on different concentrations of **DBF3.**

Table S3. Cell count after exposure to **DBF3**

Replicates	Vehicle Control wells (SFM)	0.6 mg/ml	0.3 mg/ml	0.15 mg/ml	0.075 mg/ml	0.0375 mg/ml	0.01875 mg/ml	0.009375 mg/ml	0.0046875 mg/ml
1	584536	191051	299183	374333	504535	476185	581656	433093	562343
2	553352	199559	272519	414644	479792	472534	597656	480544	550070
3	554195	192123	274452	454466	481004	496474	476726	492810	450747
4	580648	218213	290498	449241	449459	529602	432413	587529	489583
5	557952	190383	272378	449000	484382	544620	437043	568428	553187
6	574458								
7	535792								
8	557789								
9	552322								
10	567482								
**Avearge**	**561852.6**	**198265.8**	**281806**	**428336.8**	**479834.4**	**503883**	**505098.8**	**512480.8**	**521186**
**Percentage**		**35.288**	**50.157**	**76.237**	**85.402**	**89.682**	**89.899**	**91.213**	**92.762**

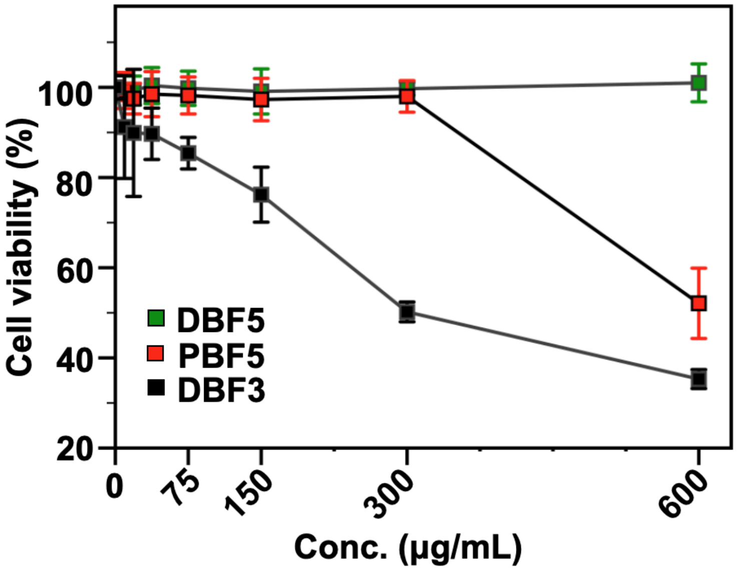

Figure S46. Comparison of cell viability of **DBF5**, **DBF3**, and **PBF5.** A549 cells were exposed to increasing concentrations (0-600 μg/mL) for 48 h.
